# Vibration‐Induced Stabilization of Lithium Anodes: Synergistic Effects of Morphological and SEI Evolution

**DOI:** 10.1002/advs.202502192

**Published:** 2025-04-15

**Authors:** Taeksoo Jung, Sunghyun Jie, Huiyeol Lee, Jinwook Jung, Joonhee Kang, Seunghun Baek, Byeongyong Lee

**Affiliations:** ^1^ School of Mechanical Engineering Pusan National University Busan 46241 Republic of Korea; ^2^ Department of Nanoenergy Engineering Pusan National University Busan 46241 Republic of Korea

**Keywords:** dendrite suppression, lithium anode, lithium metal batteries, mechanical vibration, solid electrolyte interphase

## Abstract

Lithium metal is a highly attractive anode material for next‐generation energy storage systems due to its extremely high energy density and low redox potential. Despite the growing interest in lithium metal batteries for electric vehicles (EVs), the effect of mechanical vibrations—a common condition in automotive environments—on lithium metal anodes has rarely received attention. In this study, the impact of linear shear vibration on Li metal anodes is investigated and the resulting electrochemical behavior is analyzed. Cells exposed to horizontal vibration exhibited a thinner Li electrodeposition layer compared to non‐vibrated cells (7.18 µm vs 11.3 µm). This vibration‐induced effect also delayed the increase in overpotential in Li||Li symmetric cells, extending their cycling life by up to 30%. Moreover, full cell comprising Li metal and LiFePO_4_ demonstrated enhanced stability under horizontal vibration. Physicochemical and electrochemical analyses revealed that the Li_2_O‐rich solid electrolyte interphase (SEI) formed on the electrode surface, leading to densely packed Li deposits and improved cycling performance. These findings present a novel strategy to enhance the electrochemical performance of Li electrodes through the application of linear vibration, offering valuable insights for designing EV batteries.

## Graphical abstract

This study, for the first time, highlights the marvelous effect of mechanical vibration on the performance of lithium metal batteries. The authors demonstrate that vibration enhances the performance of lithium metal anodes through a synergistic effect: surface flattening at the macroscale and the formation of an interfacial layer that facilitates fast charge transfer kinetics at the microscale.

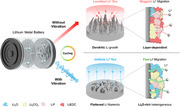

## 1 Introduction

Lithium‐ion batteries (LIBs), known for their high energy density and long cycle life, have predominantly powered electronic devices over the past decade.^[^ ^1^ ^]^ With rising concerns about climate change, the automotive industry is transitioning from internal combustion engines to LIBs in electric vehicles (EVs).^[^ ^2^ ^]^ However, as LIBs have been incorporated into EVs, their limited driving range has often become a growing concern.^[^ ^3^ ^]^ To address the issue, high‐energy electrodes have been explored extensively,^[^ ^4^ ^]^ including silicon,^[^ ^5^ ^]^ Li metal,^[^ ^6^ ^]^ Na metal,^[^ ^7^ ^]^ K metal,^[^ ^8^ ^]^ etc. Lithium metal electrodes are especially promising due to their high energy density and low electrochemical potential, potentially replacing graphite anodes to significantly improve energy density. Based on lithium metal anodes, advanced battery concepts such as Li‐S, Li‐O_2_, and Li‐High Ni have been proposed.^[^ ^9^ ^]^

To date, the majority of studies have been conducted under the assumption that batteries operate in controlled environments and have primarily focused on the design of cell components to achieve optimal performance, including electrode structure,^[^ ^10^ ^]^ and electrolyte formulations.^[^ ^11^ ^]^ However, in practical applications, batteries are inevitably exposed to various environmental conditions that can significantly influence their performance. In this context, the effects of those environmental conditions on Li‐based batteries have been investigated in a couple of aspects.^[^ ^12^, ^13^, ^14^, ^15^, ^16^, ^17^, ^18^, ^19^ ^]^ For instance, at elevated temperature increased Li‐ion diffusivity and lithiophilicity promoted the formation of larger Li nuclei with small nucleation, leading to reduced overpotential of a Li metal electrode.^[^ ^12^ ^]^ Regarding pressure as a variable, both external and internal pressure can impact the performance of lithium metal electrodes. In the case of external pressure, higher pressure helps to suppress dendrite growth, which enhances cycling stability and safety.^[^ ^13^, ^14^ ^]^ Internal pressure, generated during the electrochemical processes, is considered to improve the contact between the electrode and electrolyte, though this effect depends on the pressure distribution and magnitude.^[^ ^15^ ^]^ The effect of external factors, even in extreme conditions such as in space, has been investigated by Gao et al.^[^ ^17^, ^18^ ^]^ Supergravity up to 50 G induced an inorganic‐rich solid electrolyte interphase (SEI) layer and homogenized the surface of lithium metal electrodes.^[^ ^17^ ^]^ Meanwhile, gamma radiation in space led to a deterioration in electrochemical performance, with gamma‐exposed Li interfaces exhibiting thick SEI layers due to severe side reactions of the electrolyte.^[^ ^18^ ^]^

Since vehicles are prominent sources of vibration, batteries in such applications would be significantly affected by these forces. The impact of vibration on batteries has been partially elucidated. For example, studies have shown that vibrations can cause mechanical degradation of a cell's constituents, leading to a decrease in discharge capacity.^[^ ^20^ ^]^ Hooper et al. showed that i) the electrochemical performance of a cell, such as discharge capacity and electrochemical impedance, can noticeably deteriorate due to changes in anode surface chemistry caused by vibration, and ii) the performance is dependent on the direction of vibration.^[^ ^21^, ^22^ ^]^ Subsequent studies showed that increases in internal resistance can occur even at frequencies below 30 Hz,^[^ ^23^ ^]^ with this increased resistance attributed to changes in surface morphology due to particle agglomeration.^[^ ^24^ ^]^ However, the correlation between vibration and lithium metal batteries (LMBs) has not yet been thoroughly investigated.

Previous research has shown that vibration profiles can significantly affect battery systems.^[^ ^25^ ^]^ However, to effectively analyze and understand the impact of vibrations on LMBs, it is crucial to conduct studies focusing on individual frequency ranges. By isolating the effects of specific frequency ranges on LMBs, it becomes possible to accurately determine the influence of each frequency component. This approach allows for a more comprehensive understanding of how vibrations affect battery performance, which can then be utilized in the design and optimization of batteries to enhance their resilience and efficiency under real‐world conditions. Considering vibrations are typically prominent at low frequencies,^[^ ^25^ ^]^ understanding the behavior of Li anode under this dynamic environment is of fundamental significance.

**Figure 1:**
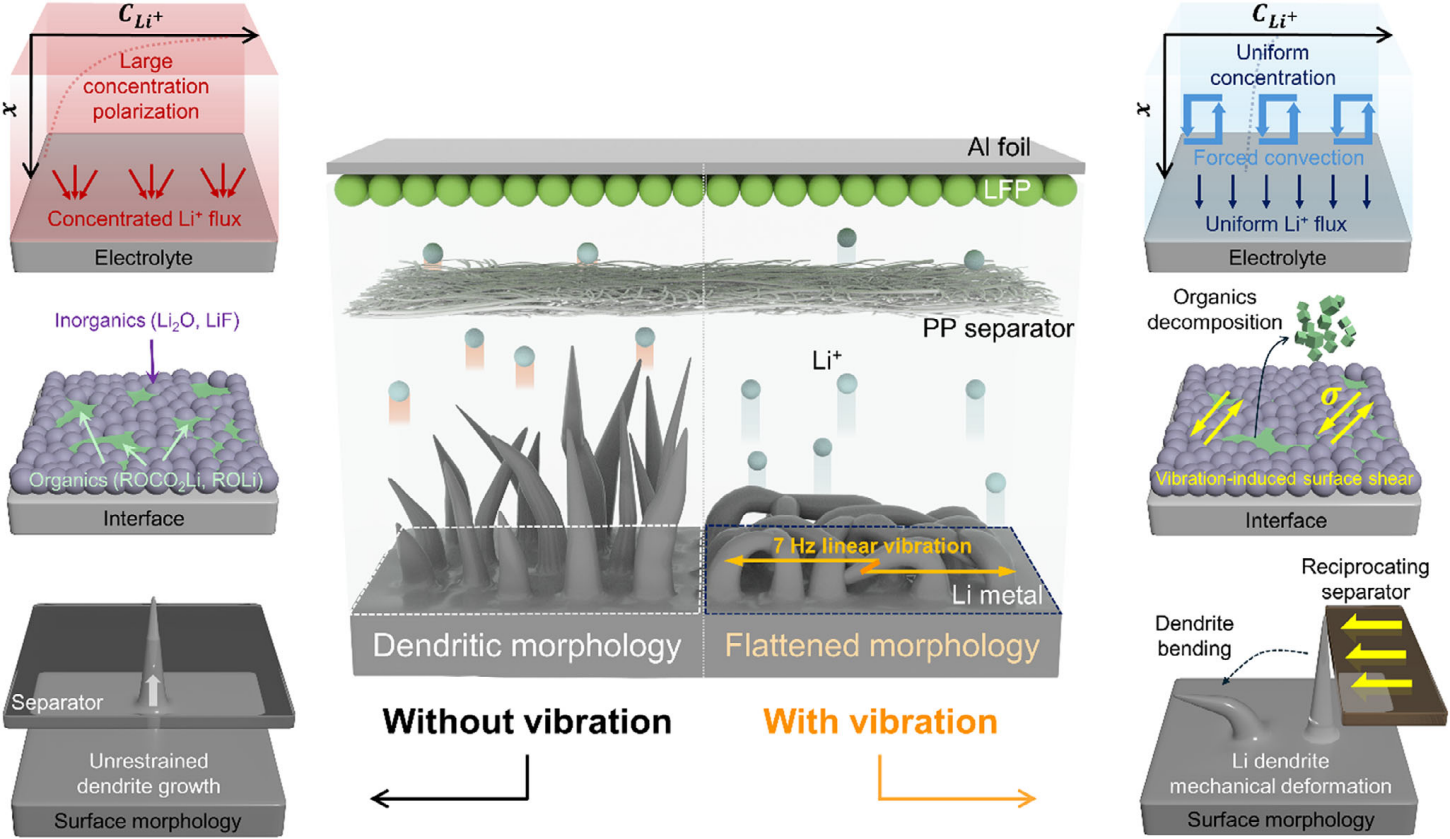
Schematic illustration of the effects of linear vibration on lithium metal anodes.

Herein, we report the significant effect of low‐frequency vibration on LMBs, especially concerning the surface of the lithium metal electrode. We imposed linear‐reciprocating vibration on lithium metal electrodes. By applying horizontal vibration in the electrode plane direction, Li||Li symmetric cells exhibited lower overpotentials (60 mV vs 90 mV after 100 cycles) and prolonged cycle life at 1 mA cm⁻^2^ and 1 mAh cm⁻^2^. As a result, when coupled with a LiFePO₄ (LFP) cathode, the Li||LFP cell also exhibited a stable cycling life of 250 cycles at 0.5C (85 mA g⁻¹) under vibration. A series of physicochemical studies revealed that the implementation of vibration induces changes in morphology and surface chemistry. With the vibration‐derived heterogeneous SEI layer, the lithium electrode exhibited a densely packed structure, resulting in enhanced electrochemical performance. Furthermore, the vibration‐induced shear force altered the surface chemistry of the lithium anode. Specifically, the ratio of Li₂O among the inorganic SEI components was increased, generating an efficient diffusion path for Li⁺ ions. Notably, this increased ratio of Li₂O in the SEI components originated from the high mechanical stability of Li₂O among other components, allowing it to withstand the shear forces caused by vibration‐induced cross‐flow (**Figure** **1**). These results indicate the viability of the application of LMBs in EVs and may open a novel avenue for research into the effects of other external forces on LMBs.

## 2 Results and Discussion

### 2.1 Coulombic Efficiency Evaluation under Linear Vibration

To explore the influence of vibration on the electrochemical performance of Li metal, Li||Cu half cells were investigated with and without vibration. The vibration was introduced through reciprocating linear motions. First, the coulombic efficiency of Li||Cu cells was calculated to assess the reversibility of the electrochemical system (Figure S1a, Supporting Information). To reduce uncertainty in coulombic efficiency with conventional methods, the average efficiency was calculated using Aurbach method.^[^ ^26^ ^]^ The cell cycled with vibration exhibited an improved coulombic efficiency of 83.8% compared to 78.2% for the cell without vibration. The higher average coulombic efficiency can be attributed to the uniform deposition of lithium and the formation of a stable solid electrolyte interphase (SEI) under horizontal vibration, resulting in more reversible plating and stripping of lithium on the copper substrate.^[^ ^27^, ^28^ ^]^ In the first cycle, cells with vibration exhibited a lower nucleation overpotential (149 mV) compared to that of cells without vibration (193 mV) (Figure S1b, Supporting Information). Additionally, the lower overpotential for the vibrated cell was maintained over the cycles (Figure S1c, Supporting Information). It is considered that nucleation overpotential is influenced by current density distribution,^[^ ^29^ ^]^ and cross‐flow between electrodes regulates electroconvection, leading to a uniform current density on the electrode surface.^[^ ^30^ ^]^ To investigate this further, we analyzed polarization behavior under vibration (Figure S2, Supporting Information). Under both horizontal and vertical vibrations, the Cu electrodes exhibited significantly higher current densities at lower overpotentials compared to the non‐vibrated case, indicating enhanced interfacial kinetics due to improved ion transport. Therefore, the reduced overpotential and enhanced lithium metal cycling might be interpreted by the effect of cross‐flow and resulting ion‐concentration homogenization within the cell, which promotes a more uniform Li^+^ flux. Notably, vertical vibration also improved Coulombic efficiency (83.3%), comparable to that of horizontal vibration (83.8%), likely due to direct Li⁺ convection that minimized concentration polarization and reduced nucleation overpotential (Figure S3a,b, Supporting Information). However, during subsequent cycles, higher voltage polarization was observed under vertical vibration (Figure S3c, Supporting Information), which may result from less effective ion transport within the porous lithium layer compared to the shear‐driven electrolyte mixing induced by horizontal vibration.

**Figure 2:**
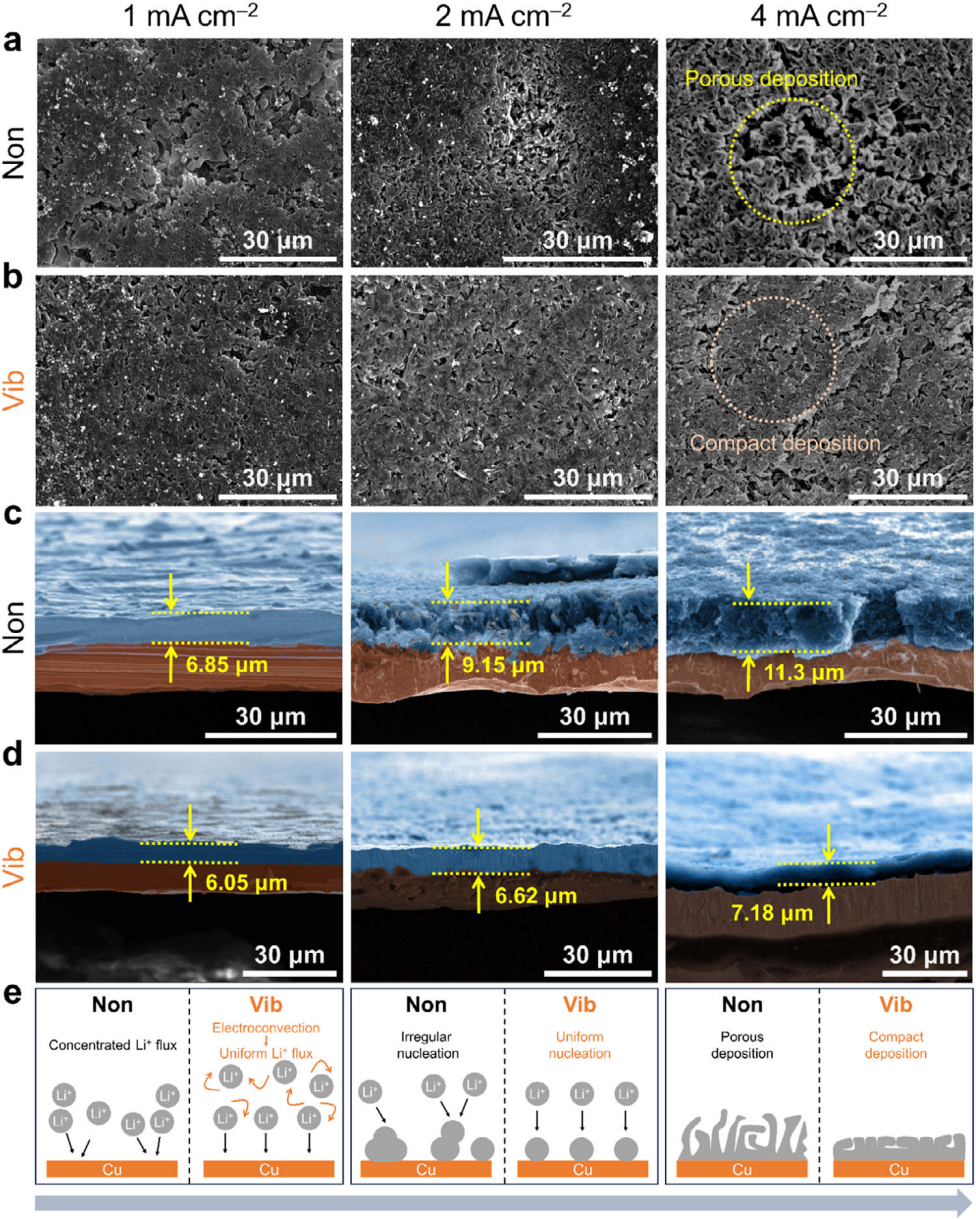
Top‐view SEM images of the Li deposition a) without and b) with vibration. Cross‐sectional SEM images of Li deposition c) without and d) with vibration. e) Schematic illustration of the Li plating with and without vibration.

To underpin the effect of vibration behind the enhanced coulombic efficiency, the morphology of Li deposited on the Cu substrate was investigated (**Figure** **2**; Figure S4, Supporting Information). Top view and cross‐sectional images of plated Li at various current densities were analyzed at an areal capacity of 1 mAh cm^−2^. It was observed that linear vibration significantly influenced the structure and thickness of deposited Li. At a low current density (1 mA cm⁻^2^) without vibration, lithium deposition appeared sparse, with visible voids and cracks between deposited regions (Figure 2a). In contrast, the morphology of deposited lithium under vibration exhibited fewer cracks and more uniformly filled areas (Figure 2b). As the current density increased, the difference in the sparsely filled areas on the surface between cells with and without vibration became more pronounced. The cross‐sectional images further illustrate the critical effect of linear vibration on the electrodeposition of lithium (Figure 2c,d). At 1 mAh cm^−2^, the thickness of deposited Li with vibration was 6.05 µm, significantly lower than that without vibration (≈6.85 µm). As the current density increased, the thickness of the electrode without vibration significantly increased from 6.85 to 11.3 µm. Consistent with the top view, the cross‐sectional view showed that Li plated without vibration was less dense and more irregular. Conventionally, based on Sand's time effect, the increase of the current density accelerates the growth of dendrites as well as the increase of overpotentials.^[^ ^31^ ^]^ However, the cells with vibration showed a small increase in thickness, from 6.05 µm at 1 mA cm^−2^ to 7.18 µm at 4 mA cm^−2^ (an 18% increase in thickness in the normal direction despite the higher current density). The compact lithium deposition under vibration highlights its beneficial role in promoting uniform and dense lithium deposition. Similar to the coulombic efficiency results, the voltage profiles at the initial cycle showed smaller values at all current densities in vibrated cells (Figure S5, Supporting Information). Normally, in carbonate electrolytes with LiPF_6_, Li dendrites usually exhibit less dense (mossy, fluffy, needle‐like) structures.^[^ ^32^ ^]^ However, as illustrated in Figure 2e, vibration appeared to inhibit dendrite growth and thus promote thin and compact layer deposition.

### 2.2 Electrochemical Behavior of Li Metal under Vibration

**Figure 3:**
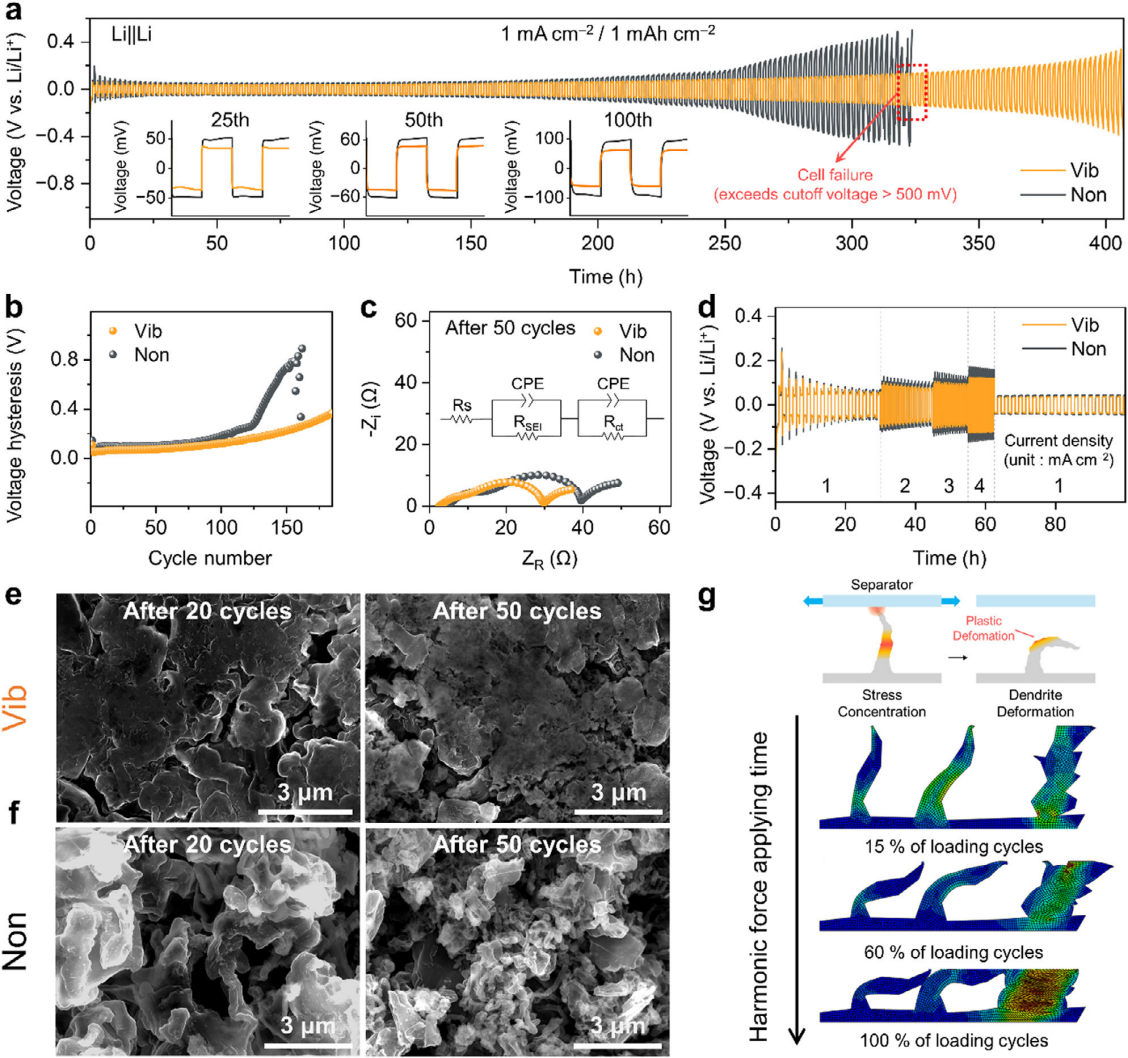
a) Cycling performance of the symmetric Li cells with and without vibration. Insets: enlarged voltage profiles at the 25th, 50th, and 100th cycles. b) Voltage hysteresis of the cells. c) Impedance measurement of the cells (the inserted equivalent circuit represents the fitted impedance result). d) Rate‐dependent performance of the cells from 1  to 4 mA cm^−2^ and reverse back to 1 mA cm^−2^. Top‐view SEM images of the Li electrodes cycled for 20 and 50 cycles e) with vibration f) and without vibration. g) Finite element analysis of the effects on harmonic vibration on lithium dendrite morphology.

To investigate the influence of vibration on cycle life, plating and stripping tests were carried out using Li||Li symmetric cells with (Vib) and without vibration (Non) at 1 mA cm⁻^2^ and 1 mAh cm⁻^2^ (**Figure** **3**a). Compared to Non, Vib showed significantly enhanced cycling stability. For lithium plating overpotential, Non exhibited a voltage of 47 mV at the 25th cycle, which continued to increase with cycling (e.g., ≈50 mV at the 50th cycle and ≈90 mV at the 100th cycle) (inset in Figure 3a). The overpotential of Non exhibited a sudden drop at ≈150 cycles (Figure S6, Supporting Information) and started to fluctuate rapidly after 160 cycles (Figure S7, Supporting Information). This oscillation may be attributed to the unstable SEI,^[^ ^33^ ^]^ eventually leading to cell failure by exceeding the cut‐off voltage of 500 mV. In contrast, Vib maintained stable voltage profiles throughout the cycle test, with overpotentials of ≈33 , 36 , and 60 mV after 25, 50, and 100 cycles, respectively (inset in Figure 3a). It is worth noting that the emergence of an arc‐shaped voltage profile, commonly observed with dead lithium accumulation,^[^ ^34^ ^]^ was delayed in Vib (Figure S8, Supporting Information). The delayed appearance may be attributed to reduced dead lithium accumulation in Vib, likely due to a densely packed deposition morphology. Figure 3b compares the voltage hysteresis between Vib and Non cells, displaying a rapid increase in overpotential for Non compared to Vib. To understand voltage hysteresis, the resistance of the cells was examined using an equivalent circuit model at varied cycles (Figure 3c). From high to low frequencies, the two semicircles in the Nyquist plots were observed, which indicates the resistance of the SEI (R_SEI_) and the charge transfer resistance (R_ct_). After 50 cycles of plating and stripping, the R_SEI_ of Vib was 6.61 Ω, which was 34% lower than that of Non (10.06 Ω). The lower R_SEI_ indicates higher ionic conductivity of the SEI layer, likely due to the difference in SEI composition in Vib. R_ct_ represents electrochemical kinetics at the interfaces. After 50 cycles, R_ct_ of Vib represented 20.65 Ω, while that of Non was 25.84 Ω, indicating that vibration indirectly affected the charge transfer. Previous studies suggest that higher R_ct_ values correlate with an unstable Li metal interface, hindering charge transfer kinetics.^[^ ^35^ ^]^ According to the voltage profiles, R_SEI_, and R_ct_ of Vib and Non, it is reasonable that the vibration‐induced stable SEI has a faster charge transfer kinetics and enhances the cycling stability of cells as well. Additionally, Vib exhibited higher exchange current density compared to Non in Tafel plots (Figure S9, Supporting Information). The enhanced exchange current density in Vib further confirms that vibration‐induced SEI facilitates ion transfer reaction at the interface of electrodes.^[^ ^36^ ^]^ To further investigate the performance of lithium metal electrodes under vibration, a rate capability test was performed from 1 to 4 mA cm⁻^2^ with a fixed areal capacity of 1 mAh cm⁻^2^ (Figure 3d). Vib exhibited a consistently lower overpotential than Non, with the difference becoming more pronounced as the current density increased. The improved electrochemical stability was also evident in cycling tests at increased current densities (2 and 4 mA cm⁻^2^) (Figure S10, Supporting Information). Figure 3e,f shows the surface morphology of cycled Li electrodes with and without vibration at a current density of 1 mA cm^−2^ with an areal capacity of 1 mAh cm^−2^. For Vib, the deposited Li exhibited dense, flattened structures (Figure 3e). This flattened morphology was retained even afer 100 cycles (Figure S11, Supporting Information), which is favorable to uniform lithium deposition.^[^ ^37^ ^]^ Conversely, a sparse and fibrous morphology was found in Non as reported previously (Figure 3f). As cycling continued, numerous gullies formed on the surface, and the irregularity became severe (Figure S12, Supporting Information), potentially deteriorating electrode stability.^[^ ^38^ ^]^ The cross‐sectional view of the electrode more clearly shows the effect of vibration on the morphology (Figure S13, Supporting Information). After 100 cycles, the Li electrode cycled under vibration retained a dense and uniform morphology, whereas the non‐vibrated electrode exhibited a porous, and uneven structure with pronounced voids. Overall, the surface morphology and electrochemical characterization suggest that vibration induces dense bulk deposition morphology, lowering resistance and boosting fast kinetics.

### 2.3 Intrinsic Mechanism of Morphology Evolution under Linear Vibration

The difference in deposition morphology could be understood by analyzing the behavior of Li dendrites under tangential loading, applying beam theory with appropriate modifications to account for the irregular geometry of dendrites. In an ideal cantilever beam, plastic deformation initiates at the surfaces near the root when loading exceeds the yield point, progressing inward and along the beam length. This phenomenon, known as “plastic hinge” formation, leads to stress redistribution across the beam section. For a rectangular beam section, the plastic moment capacity (*M_p_*) is given by:

(1) $$M_{p}=\sigma_{y}\;Z=\sigma_{y}\;\frac{bh^{2}}{4}$$

where *Z* is the plastic section modulus, *σ_y_* is the yield stress of the material, and b and h are the width and depth of the cross‐section, respectively. In the case of an ideal tapered cantilever beam, the maximum stress under tangential loading typically occurs at the fixed end (root) of the beam since the bending moment is maximum at the fixed end, decreasing linearly toward the free end. Additionally, the abrupt change in geometry at the fixed end creates a stress concentration effect. Consequently, normal stresses due to bending and shear stresses due to the applied load combine to create a complex stress state. It's important to note that while the maximum stress typically occurs at the root, the exact stress distribution can be influenced by factors such as the taper angle and the area of the cross section.

To overcome the limitations of analytical methods in describing complex geometries, finite element analysis (FEA) was employed to reveal the geometric changes of dendrites under horizontal vibration. A 2D FEA model was constructed based on experimental observations, with an average dendrite thickness of 1.5 µm, Young's modulus ranging from 1.6 to 2.3 GPa, and a density of 2 g cm⁻^3^.^[^ ^39^, ^40^ ^]^ The FEA simulations demonstrated that relative motion induces flexural stress within the dendrite, leading to bending deformation as the loading cycle progresses. Unlike ideal tapered cantilever beams where maximum stress typically occurs at the fixed end, dendrites exhibit a more complex stress distribution due to their irregular cross‐sections. As shown in Figure 3g, stress can be localized in the middle of the dendrite where the geometry changes abruptly. Regions experiencing the highest bending moments are more susceptible to plastic deformation, with cumulative plastic strain causing further bending over successive loading cycles. The FEA results indicate that horizontal vibration mechanically conditions the Li surface by flattening dendritic Li. The combination of experimental observations and finite element analysis provides valuable insights into the behavior of lithium dendrites under tangential loading.

### 2.4 Effect of the Linear Vibration on the SEI Structure

**Figure 4:**
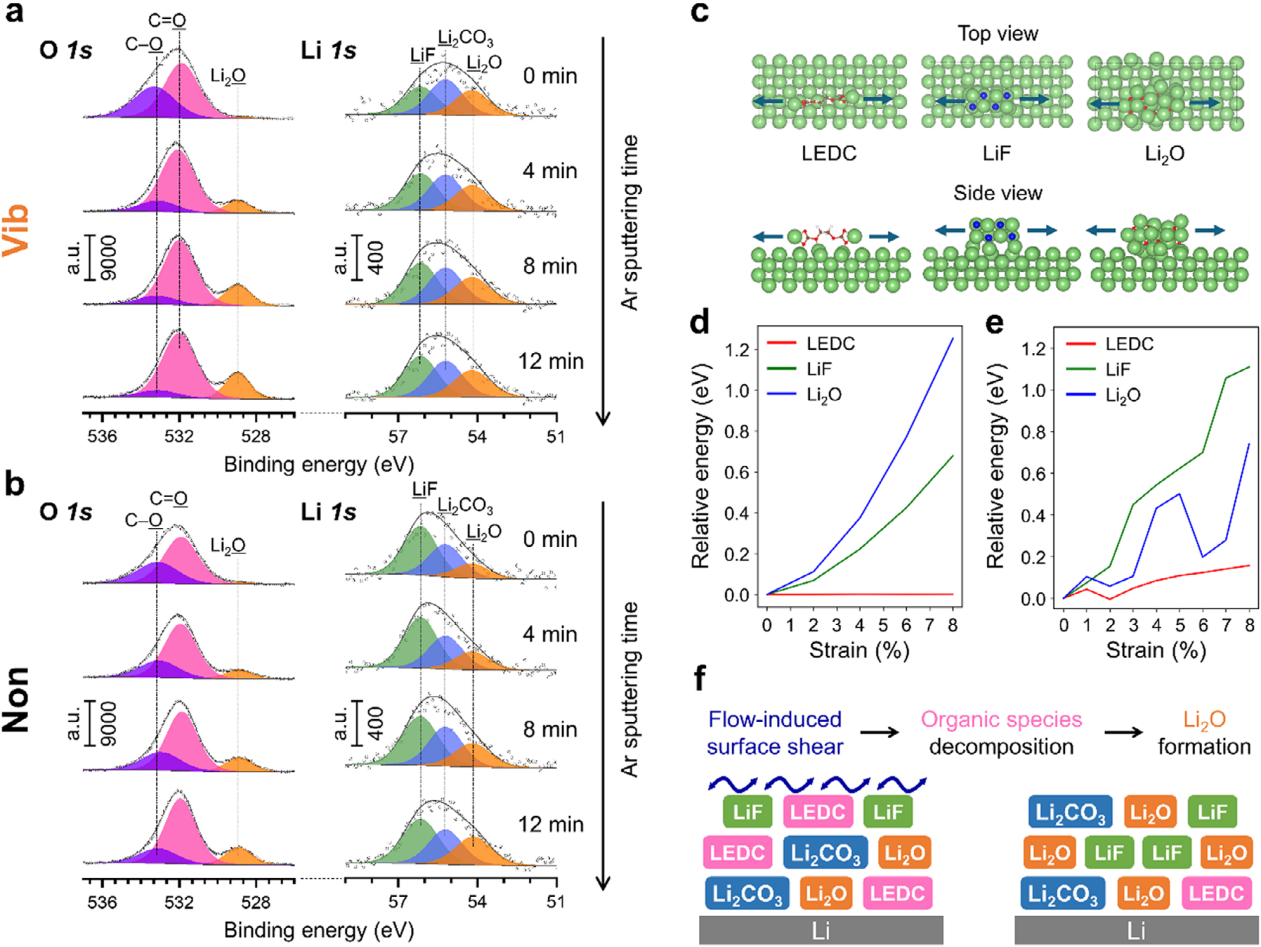
XPS spectra of the Li anode cycled a) with vibration and b) without vibration after various sputtering times: 0, 4, 8, and 12 min. c) Configurations of the different SEI components on the top of Li anode for calculation. H, Li, C, O, and F are shown in white, green, brown, red, and blue colors, respectively. Comparison of the relative energy of LEDC, LiF, and Li_2_O as a function of strain: d) bulk condition and e) interface condition. f) Schematic illustration showing the conversion of organic species into Li_2_O due to vibration‐induced cross flow.

To address the enhanced Li kinetics and dendrite suppression of Vib, the chemical composition of cycled Li electrodes with and without vibration was analyzed using in‐depth XPS measurements (**Figure** **4**). High‐resolution O 1s spectra of Vib and Non were fitted with three curves: C ‐ O (533.1 ± 0.2 eV), C = O (531.6 ± 0.2 eV) and Li_2_O (528.9 ± 0.1 eV) (Figure 4a,b).^[^ ^17^ ^]^ Both Vib and Non displayed C = O and C–O peaks before etching. After etching, Li_2_O appeared in both samples, with a relatively higher Li_2_O content in the horizontally vibrated sample compared to Non. To further investigate the inorganic components of the SEI layer, high‐resolution Li 1s spectra were fitted with three curves: LiF (56.2 ± 0.2 eV), Li_2_CO_3_ (55.2 ± 0.1 eV) and Li_2_O (54.2 ± 0.1 eV) with a full width at half maximum (FWHM) of 1.5 ± 0.1 eV (Figure 4a,b).^[^ ^41^ ^]^ The ratios of these species in the Li 1s spectra for Vib and Non are shown in Figure S14 (Supporting Information). Overall, the surface layer on the Vib electrode exhibited a heterogeneous structure consisting of LiF, Li_2_CO_3_, and Li_2_O. In contrast, the Non electrode surface contained more LiF on the outer layer, becoming more heterogeneous (with LiF, Li_2_CO_3_, Li_2_O mixed) as the etching time increased. Additionally, high‐resolution C 1s spectra were fitted by curves of C‐C/C‐H (284.7 ± 0.3 eV), C‐O (285.6 ± 0.2 eV), C = O (288.8 ± 0.1 eV), and Li_2_CO_3_ (290.1 ± 0.1 eV) (Figure S15, Supporting Information).^[^ ^17^ ^]^ C‐C/C‐H, C‐O, and C = O component peaks are related to organic compounds derived from electrolyte decomposition.^[^ ^42^ ^]^ There was no significant difference between the Vib and Non samples at different etching times, suggesting that the overall organic composition of the SEI layer remained similar under both conditions. The observed similarity in C 1s spectra indicates that while the horizontal vibration mostly influences the inorganic components of the SEI, it does not markedly alter the organic composition.

Li_2_O is considered a representative inorganic SEI component with a low Li^+^ diffusion barrier, favorable for the suppression of dendrites.^[^ ^43^ ^]^ In terms of Li^+^ transport in the SEI, the diffusion coefficient at the LiF/Li_2_O grain boundary is higher than at the LiF/LiF boundary.^[^ ^44^, ^45^, ^46^ ^]^ This improved diffusion coefficient of Li_2_O is attributed to multiatom coordination of Li, leading to a multiatom hopping mechanism that is more efficient than other mechanisms.^[^ ^47^ ^]^ Therefore, the heterogeneous SEI layer in the horizontally vibrated cells, particularly with a balanced ratio of Li_2_O and LiF, is believed to facilitate faster Li^+^ kinetics, enhancing the electrochemical performance and stability of the Li metal electrodes.

To understand the changes in SEI composition, we systematically investigated the behavior of typical SEI components under vibration. We modeled three representative SEI components—dilithium ethylene dicarbonate ([CH_2_OCO_2_Li]_2_, LEDC), LiF, and Li_2_O—on the Li surface and applied tensile stress from flow‐induced surface shear force to these species (Figure 4c). A comparison of their resistance to deformation showed that Li_2_O exhibited the highest resistance, followed by LiF, while LEDC demonstrated the weakest resistance (Figure 4d). This trend aligns with the high Young's modulus of Li_2_O and LiF, which are 169 and 64.9 GPa, respectively, compared to the low modulus of organic components like ROCO_2_Li/ROLi, which is less than 1 GPa.^[^ ^48^ ^]^ Under vibration‐induced stretching, LEDC displayed instability, while LiF and Li_2_O remained relatively stable (Figure 4e). Specifically, for Li_2_O, it was observed that when 2% and 6% strain was applied to the Li metal surface, the energy became more stable, and subsequent structural changes became more difficult. These results indicate that weak organic species decompose under vibration‐induced shear force, whereas the inorganic components are more resistant to decomposition owing to their superior mechanical properties (Figure 4f).

### 2.5 Influence of Vibration Parameters on Electrochemical Behavior

Building upon the favorable electrochemical performance observed under horizontal vibration, we further evaluated how varying vibration parameters, including direction, amplitude, and frequency, affect the behavior of Li metal electrodes. In the case of vertical vibration, it was observed that the Li deposition on Cu was more uniform compared to non‐vibrated conditions, but less effective than horizontal vibration (Figure S16a, Supporting Information). This trend appeared not only in the deposition morphology but also in nucleation overpotential—vertical vibration led to improved performance over Non yet remained less effective than horizontal vibration (Figure S16b, Supporting Information). A similar moderate effect was observed in the cycling stability of Li||Li cells at various current densities (Figure S17, Supporting Information). Under vertical vibration, Li metal exhibited a favorable deposition morphology, though slightly inferior to that achieved with horizontal vibration (Figure S18, Supporting Information). This can be attributed to localized fluid motion induced by vertical vibration, which helps mitigate Li⁺ concentration gradients near the electrode surface during the early stages of cycling. However, once a porous and void‐rich Li layer is formed, horizontal vibration more effectively promotes electrolyte mixing by penetrating interparticle voids, thereby enhancing ion transport and cycling stability. Specifically, the superior electrochemical behavior with horizontal vibration implies that additional mechanisms, such as surface thinning induced by shear vibration, may contribute to its enhanced performance beyond mere convection.

We also investigated how vibration intensity, determined by amplitude and frequency, influences Li deposition behavior. SEM analysis revealed that introducing vibration transformed the initially fibrous Li morphology into a more uniform, oval‐shaped structure, which is favorable for homogeneous Li growth (Figure S19, Supporting Information). This morphological change became more pronounced at higher vibration intensity. Notably, at doubled frequency, Li deposits appeared significantly denser and more compact, suggesting that increased shear force further promotes favorable morphology. This improvement is attributed to enhanced convection at higher vibration intensity, which facilitates ion mass transfer and reduces Li⁺ concentration gradients at the electrode surface.

Additionally, XPS analysis revealed that the content of Li₂O—a mechanically stable inorganic SEI species—increased under both doubled amplitude and frequency, with the effect more pronounced at doubled frequency (Figure S20, Supporting Information). This trend aligns with the calculated shear force exerted on the electrode surface, expressed as:

(2) $$F_{max}=mA{\left(2\pi f\right)}^{2}$$

where *F* = force, *m* = mass, *a* = acceleration, *A* = amplitude of sinusoidal vibration, and *f* = frequency. Based on this relationship, doubling the frequency results in a 4‐fold increase in force, while doubling the amplitude leads to a 2‐fold increase. We propose that this increased shear force promotes the decomposition of mechanically weaker organic SEI species, while Li₂O remains intact and accumulates, due to its high mechanical stability. This process leads to the formation of a Li₂O‐enriched SEI, which contributes to interface stabilization and enhanced electrochemical performance. These results highlight that vibration parameters including direction, amplitude, and frequency synergistically influence both Li deposition morphology and SEI composition, offering valuable insights into a promising strategy to enhance the performance of LMBs through mechanically induced interface engineering.

### 2.6 Full Cells Assessment under Linear Vibration

**Figure 5:**
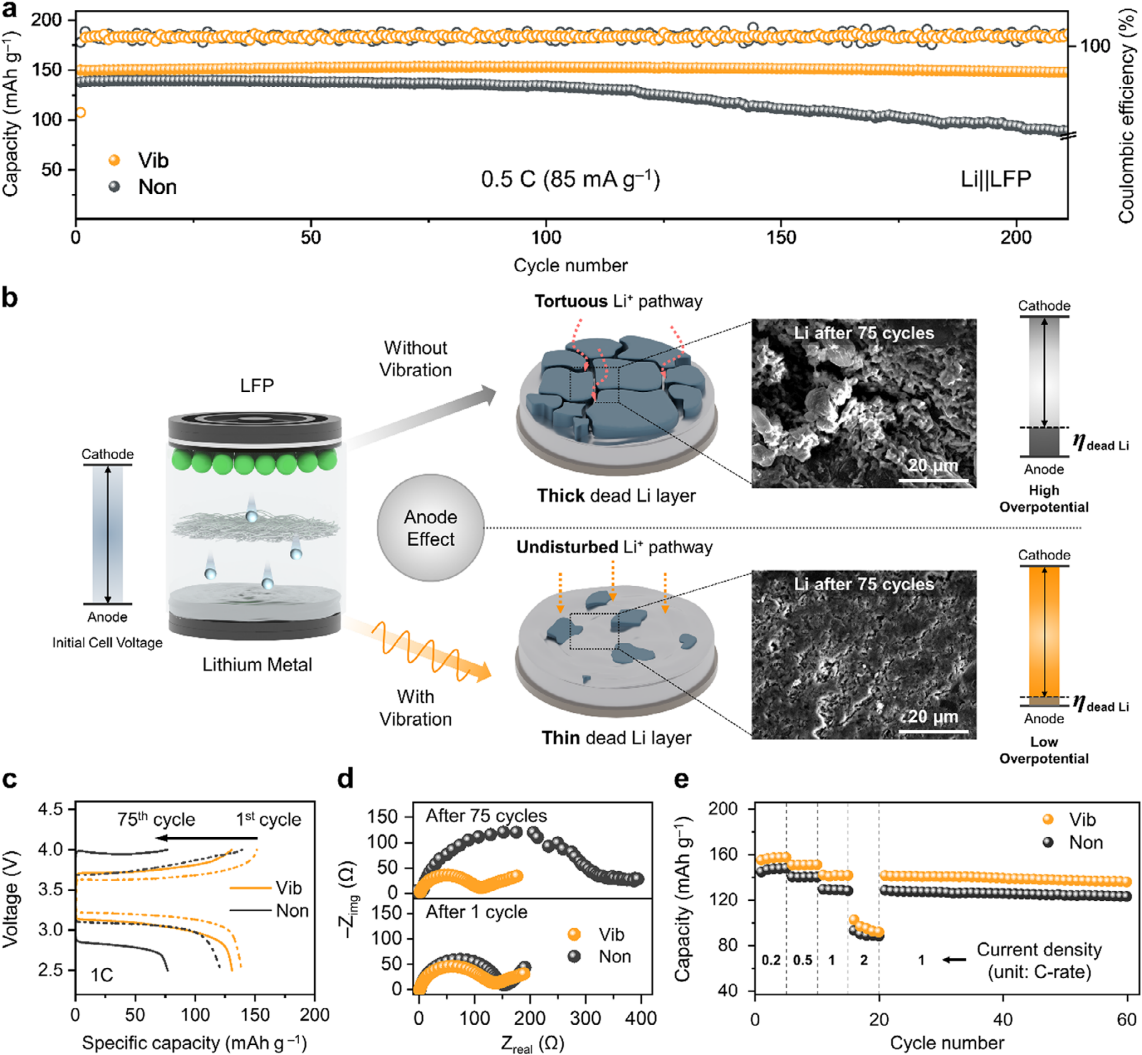
a) Cycling performance of the Li||LFP cell as a function of cycle number in the voltage range of 2.5–4.0 V at 0.5C (85 mA g^−1^). b) Schematic illustration and top‐view SEM images of Li morphology after 75 cycles at 1 C with and without vibration. c) Charge/discharge profiles at 1st and 75th cycle. d) Nyquist plots of Li||LFP cells after 1 cycle and 75 cycles with and without vibration. e) Rate performance of Li||LFP cells with and without vibration.

The effect of vibration on the cycling stability of LMBs was examined using an LFP cathode in a coin cell configuration (**Figure** **5**a; Figure S21, Supporting Information). At a 0.5 C rate (85 mA g^−1^), Li||LFP full cells exhibited increased discharge capacity and extended cycle life under horizontal vibration. In the early cycles, Vib delivered a higher capacity of up to 149 mAh g^−1^, while that of Non exhibited only 138 mAh g^−1^. In addition, Vib exhibited outstanding capacity retention of 99.1% after 200 cycles at a 0.5 C rate. However, Non experienced a substantial capacity drop of 68%, from 138 to 94 mAh g^−1^ after 200 cycles. The Ragone plot further highlights Vib's higher energy density and a slower degradation per cycle (Figure S22, Supporting Information). The cycling stability of full cells was also evaluated at a 1C rate (Figures S23 and S24, Supporting Information). Even at the higher current density, the effects of vibration were evident, as Vib showed increased discharge capacities and extended cycle life. After the cycling test, cells were disassembled to investigate the morphology of the Li metal electrodes (Figure 5b). Vib exhibited a flat morphology after 75 cycles while a rough surface was observed in Non, indicating the non‐uniform dendrite growth leading to severe dead Li accumulation. The large‐scale image also supports that Li electrodes of Vib exhibited a flattened surface while that of Non displayed a rough surface (Figure S25, Supporting Information). In galvanostatic charge‐discharge (GCD) profiles (Figure 5c), Vib exhibited smaller voltage gaps between charge and discharge plateaus compared to Non, indicating that vibration lowered overpotential during cycling. As the cycles progressed, this plateau difference became increasingly pronounced in Non, likely due to overpotential arising from significant morphological evolution and dead Li accumulation on the anode. The Li||LFP cells were further investigated by EIS (electrochemical impedance spectroscopy) to analyze the cells’ degradation during the cycling tests (Figure 5d; Figure S26, Supporting Information). The Nyquist plots were fitted by the equivalent circuit in Figure S27 (Supporting Information), with results summarized in Table S1 (Supporting Information). After the first cycle, Vib exhibited lower resistance of SEI and charge transfer (35.0 and 100.6 Ω, respectively) compared to that of Non (37.7 and 143.0 Ω, respectively) (Figure 5d). During cycling, the gap in interface resistances (R_Interface_: R_SEI_ and R_ct_) between Vib and Non widened progressively (Figure S26 and Table S1, Supporting Information). Vib showed a barely increased R_Interface_ of up to 5.45% from the 5th cycle to the 50th cycle while a significant rise of 73% was observed in Non. After 75 cycles, Vib presented a much lower R_Interface_ than Non (Figure 5d). The rate capability test was also performed to evaluate the effect of vibration on the electrochemical performance of Li||LFP cells at various C‐rates (Figure 5e). At 0.2C, 0.5C, 1C, and 2C, Vib delivered reversible capacities of 157, 150, 141, and 95 mAh g⁻¹, respectively, while Non exhibited 147, 140, 128, and 88 mAh g⁻¹ at the same C‐rates. Due to the extended plateaus in GCD profiles, Vib exhibited enhanced rate performance compared to Non (Figure S28, Supporting Information).

To determine whether mechanical vibration affects the structure and chemistry of LFP cathode, the crystallinity of LFP after cycled with and without vibration was compared (Figure S29, Supporting Information). Both electrodes showed no difference in XRD peaks, indicating that the vibration conditions used in this study preserved the structural integrity of the cathode. Additionally, SEM images comparing the LFP showed that the size and distribution of cathode particles remained unchanged (Figure S30, Supporting Information). The surface chemistry of the LFP was also investigated to assess any potential impact of vibration on the surface composition (Figure S31, Supporting Information). XPS spectra comparing the LFP showed no significant changes. These results indicate that the enhanced cycling stability of Li||LFP under vibration originates from improved stability on the anode side, rather than other factors. This underscores the role of vibration in stabilizing the Li anode.

## 3 Conclusion

In this study, we have demonstrated the significant impact of horizontal linear vibration at low frequencies on the electrochemical performance of LMBs, particularly focusing on lithium metal anodes. The application of horizontal vibration resulted in suppressed dendrite growth and a smooth Li electrode surface. Specifically, the thickness of deposited lithium decreased from ≈6.85  to 6.05 µm at 1 mA cm⁻^2^, highlighting the role of vibration in promoting dense and compact lithium deposition. FEA provided insights into the mechanical behavior of lithium dendrites under horizontal vibration. The simulations indicated that horizontal vibration induces flexural stress within the dendrites, leading to bending deformation and flattening of dendritic structures. Moreover, surface chemistry revealed that horizontal vibration affected the inorganic components of the SEI layer. The vibrated electrodes exhibited a heterogeneous Li₂O‐rich SEI, which has superior mechanical stability and favorable Li⁺ transport properties. Benefiting from the vibration‐induced SEI, Li||Li symmetric cells achieved a 30% longer lifespan at 1 mA cm^−2^ and an areal capacity of 1 mAh cm^−2^. In Li||LFP full cells, vibration improved both capacity and cycling stability at 0.5C and 1C rates.

Although further optimization of key parameters, such as vibration frequency, amplitude, and cell packaging is required to fully harness vibration as a beneficial environmental factor, this work offers a new perspective on incorporating environmental factors into battery applications, particularly for next‐generation batteries in EVs. While the positive effect of linear vibration on LMBs is clearly demonstrated, further studies are needed to explore the relationship between battery structures and vibration effects.

## 4 Experimental Section

### Vibration Imposition

The vibration was applied at a frequency of 7 Hz, which may occur while driving EVs and exist in the battery vibration tests (Figure S32, Supporting Information). A reciprocating machine with a homemade jig was used to investigate the effect of vibration during cycling tests (Figure S33, Supporting Information). Two directional vibrations were applied (horizontal and vertical) by rotating the jig 90°.

### Electrochemical Measurements

CR2032 coin cells were assembled in an Ar‐filled glove box with the content of H_2_O and O_2_ below 0.01 ppm. For Li||Cu half cells, Li metal foils (thickness 0.75 mm) were punched into 12 mm discs and polished with a scraper to eliminate impurities on the surface. The Cu foils were punched into 15 mm discs, immersed in 1 m HCl for 10 min, and rinsed separately with deionized water and acetone several times. The Cu foils were dried in a vacuum oven at room temperature immediately after rinsing. For Li||Li symmetric cells, Li metal chips with a diameter of 14 mm were used as electrodes. 120 µ*l* of 1.0 M LiPF_6_ in EC/DEC/DMC (1:1:1, by volume) was used as the electrolyte in each cell. The Li||LFP cells were cycled in galvanostatic mode within the voltage range of 2.5–4.0 V. Before cycling, cells underwent an aging process for 24 h and cycled under 0.1 C (20 mA g^−1^) for 3 cycles. The mass loading of LiFePO_4_ (LFP) is 8.3 mg cm^−2^ with a thickness of 50 µm and a diameter of 12 mm. The electrolyte was 1.0 m LiPF_6_ dissolved in a 1:1:1 volume ratio of EC/DEC/DMC with 5 wt% fluoroethylene carbonate (FEC). All coin cells were cycled in an environmental chamber to eliminate the effect of temperature gradient. The test of the cells with and without vibration was carried out on the assembled vibration jig. To increase the reliability of the study, each electrochemical test was performed multiple times. Tafel curves and electrochemical impedance spectroscopy (EIS) were obtained using a ZIVE SP1. Tafel curves of Li||Li symmetric cells were examined at a scan rate of 1 mV s^−1^ from −0.2  to 0.2 V (vs Li/Li^+^). EIS measurements were carried out in the frequency range from 50 mHz to 1 MHz with an amplitude of 10 mV.

### Material Characterization

A scanning electron microscope (SEM, VEGA, TESCAN, and Gemini 500, ZEISS) was employed to obtain the deposition morphology of lithium with and without vibration. All samples were dried and loaded on the sample stage in the Ar‐filled glove box. The sample stage was sealed in the transfer chamber and quickly loaded into the SEM chamber to minimize air exposure of lithium metal. The sample was observed with a 10 keV acceleration voltage of the electron beam. X‐ray photoelectron spectroscopy (XPS, K‐Alpha, ThermoFisher Scientific) was performed to investigate the chemical composition of the cycled lithium metal electrode. To avoid exposure of specimens to air, a vacuum transfer vessel was employed.

### Mechanical Simulation

A surrogate dendrite FEA model was developed to validate the geometric morphing of dendritic structures under mechanical loading. This model incorporated three representative geometries of dendrite structures and external excitation environments, serving as an analog for contemplated dendrites to assess the influence of mechanical vibration on flattening dendritic Li. The 2D FEA model was constructed using ANSYS APDL, a commercial FEA program. PLANE183 elements were selected for the dendrite FE model due to their capability to model 2D solid structures with two DOFs in the x‐y directions at each node. These higher‐order 2D 6‐node elements exhibit quadratic displacement behavior, allowing for better capture of nonlinear deformations, including large deflection and strain.

Mesh size was precisely controlled to ensure solution convergence. Static analysis was iteratively performed with decreasing mesh sizes until the subsequent analysis solution converged. To improve computational efficiency, mesh sizes were controlled for two distinct regions: the roots of the dendrite and the remaining area. A higher density mesh was employed near the dendrite roots. Material properties assigned to the dendrite model included Young's modulus of 2.0 GPa, Poisson's ratio of 0.3, and density of 2 g/cm^3^. Upon model completion, time domain analysis was specified using the transient analysis option with the Newmark time integration method. Considering solution convergence for large deformation analysis, the time step was defined as 1/200 of the excitation frequency. The NLGEOM option was enabled to account for large deformation effects, allowing ANSYS to recalculate the stiffness matrix at each iteration. This approach ensures more accurate modeling of structures undergoing significant shape changes during loading. Prior to analysis execution, external loading conditions were defined. Normal pressure on the dendrite was set sufficiently small to prevent vertical deformation, while harmonic motion in the tangential direction was applied to the Cu layer at 7 Hz to simulate mechanical vibration, replicating experimental conditions. This comprehensive FEA model provides a robust platform for investigating the morphological changes in dendritic structures under various mechanical loading scenarios.

### Density Functional Theory Computation

The Vienna Ab‐initio Simulation Package (VASP)^[^ ^49^, ^50^, ^51^ ^]^ was utilized to conduct first‐principles DFT calculations to study strain effect of SEI components. The exchange‐correlation interactions were represented using the Perdew–Burke–Ernzerhof (PBE)^[^ ^52^ ^]^ functional, based on the Generalized Gradient Approximation (GGA). A plane wave energy cutoff of 400 eV was applied, and the Projector Augmented Wave (PAW) method,^[^ ^53^ ^]^ a type of pseudopotential approach, was used to represent the potential field generated by core electrons acting on outer electrons. An energy convergence threshold of 10^−4^ eV was set, with the force convergence criterion kept at 0.05 eV Å^−1^ throughout the calculations. The Gamma 1×1×1 k‐point was selected for the geometric optimization of the interface structure between Li metal and SEI components.

## Conflict of Interest

The authors declare no conflict of interest.

## Supporting information



Supporting Information

## Data Availability

The data that support the findings of this study are available from the corresponding author upon reasonable request.
