# Supplementary material for: Vibration‐Induced Stabilization of Lithium Anodes: Synergistic Effects of Morphological and SEI Evolution
Source: Adv Sci (Weinh). 2025 Apr 15;12(26):2502192. doi: 10.1002/advs.202502192 (PMC12245104; doi:10.1002/advs.202502192)
Supplement: Supplementary file 1 — Supporting Information [file ADVS-12-2502192-s001.docx]

Supporting Information

**Vibration-Induced Stabilization of Lithium Anodes: Synergistic Effects of Morphological and SEI Evolution**

*Taeksoo Jung^1^, Sunghyun Jie^1^, Huiyeol Lee^2^,* *Jinwook Jung^1^, Junhee Kang^2,*^, Seunghun Baek^1,*^, and Byeongyong Lee^1,*^*

^1^School of Mechanical Engineering, Pusan National University, Busan 46241, Republic of Korea

^2^Department of Nanoenergy Engineering, Pusan National University, Busan 46241, Republic of Korea

*Corresponding authors:

E-mail: j.kang@pusan.ac.kr (J. K.); baeksh@pusan.ac.kr (S. B.); blee1015@pusan.ac.kr (B. L.)

**
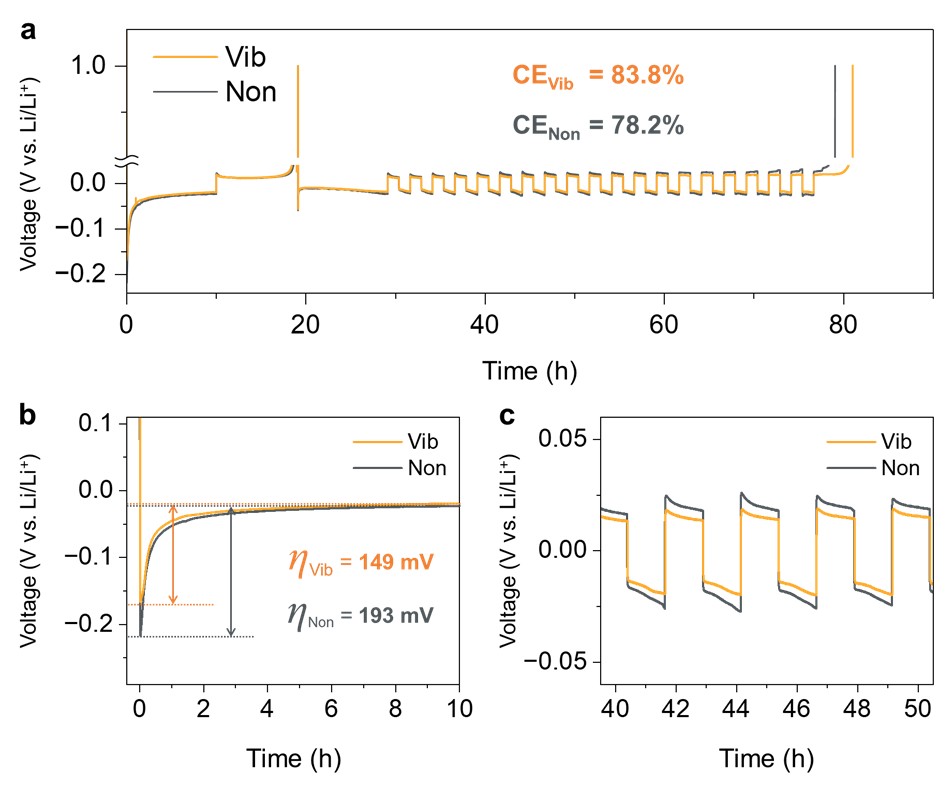
Figure S1.** Li plating/stripping performance of Li||Cu half cell with and without vibration. **(a)** Average CE determined by the Aurbach method. **(b)** Voltage profiles during initial Li plating. **(c)** Representative voltage profiles of the Li||Cu cells.

**
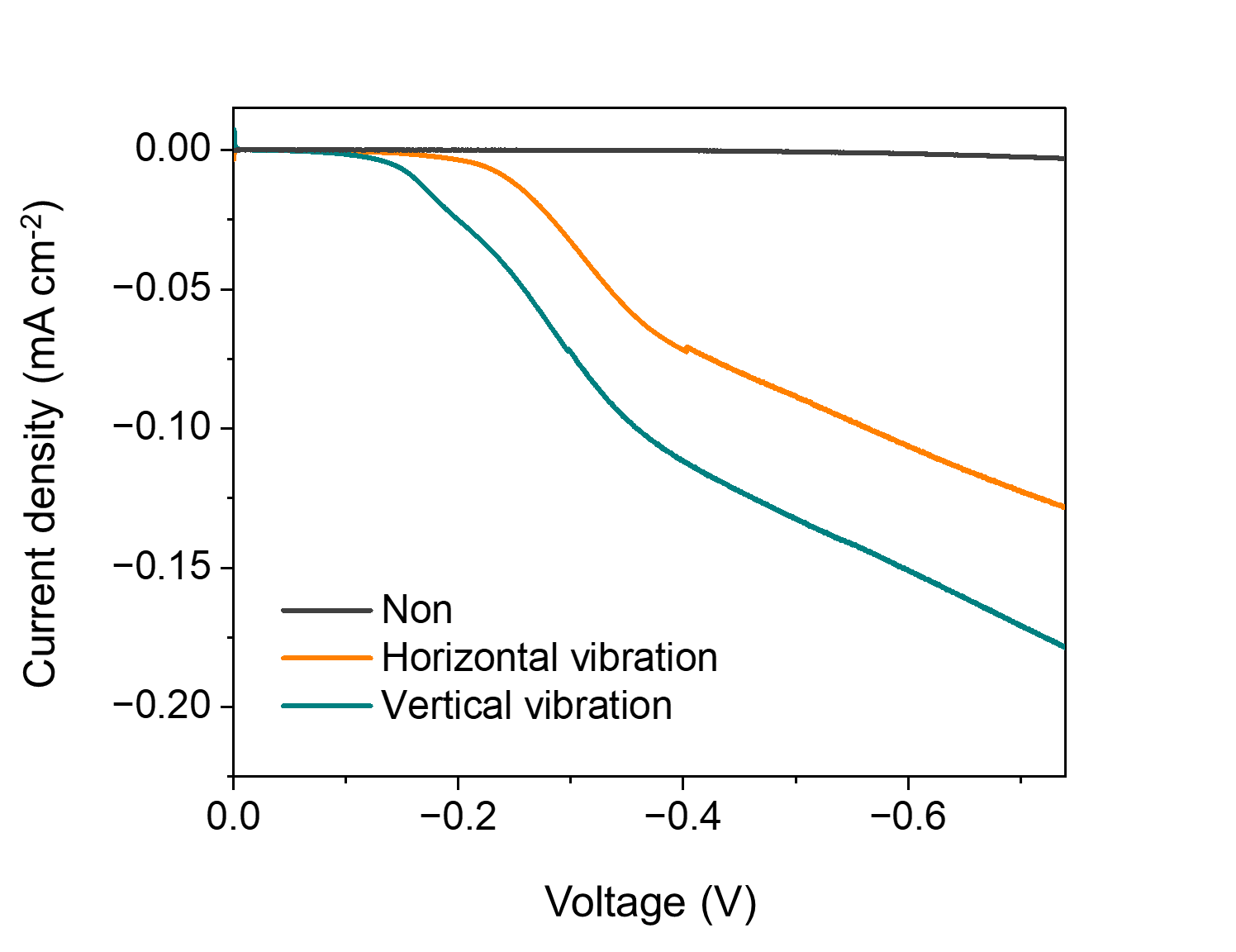
**

**Figure S2.** Polarization curves of Cu electrodes with 1M LiPF_6_ EC/DEC electrolyte measured at a scan rate of 0.2 mV s^-1^.

**
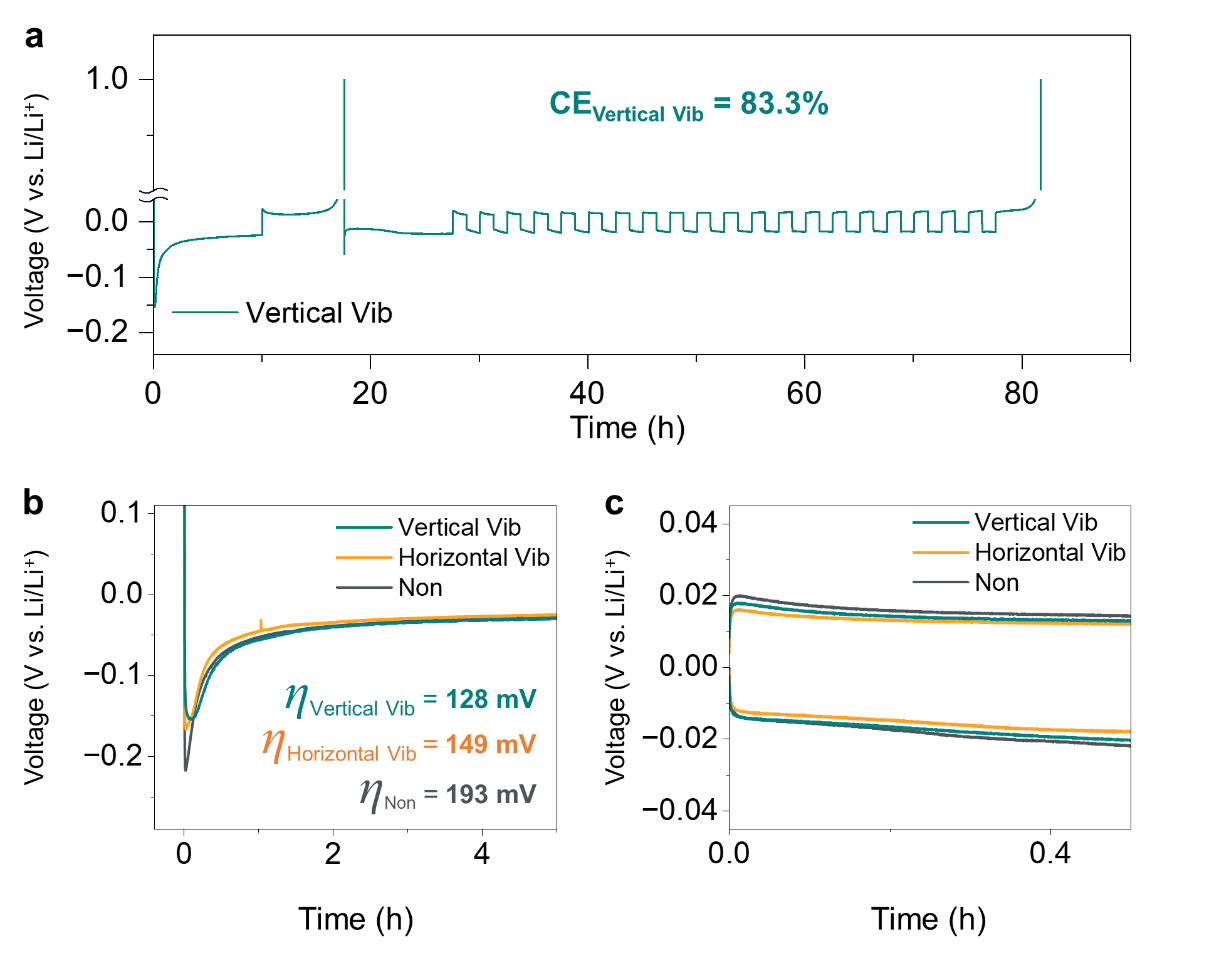
**

**Figure S3.** Li plating/stripping performance of Li||Cu half cell with and without vibration (Horizontal and vertical direction). **(a)** Average CE determined by the Aurbach method. **(b)** Voltage profiles during initial Li plating. **(c)** Representative voltage profiles of the Li||Cu cells during plating and stripping. Note: Horizontal and vertical refer to directions parallel and perpendicular to the electrode surface, respectively.

**
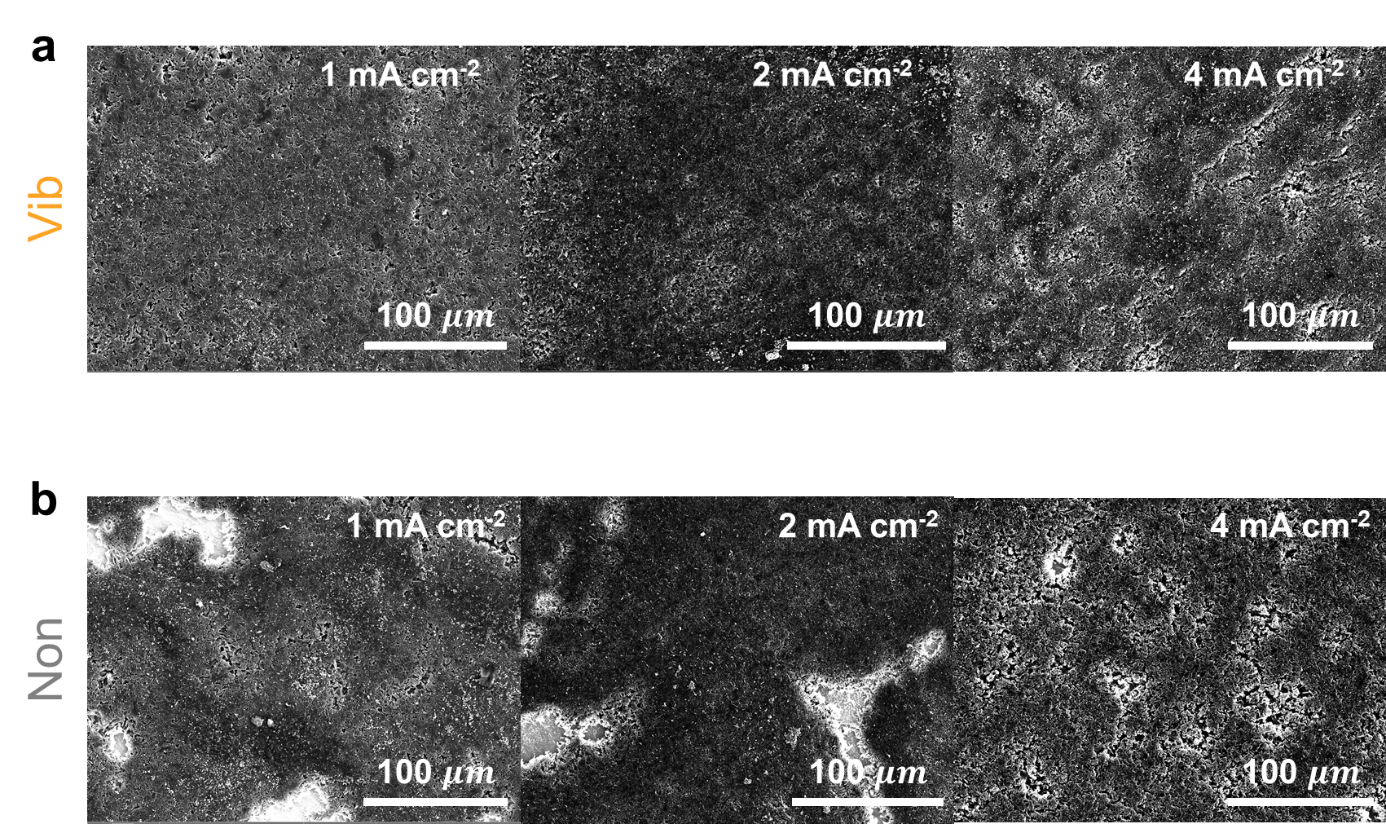
**

**Figure S4.** Top-view SEM images of the Li plated on Cu foil **(a)** with and **(b)** without vibration at different current densities of 1 mA cm^–2^, 2 mA cm^–2^, and 4 mA cm^–2^ with an areal capacity of 1 mAh cm^–2^.


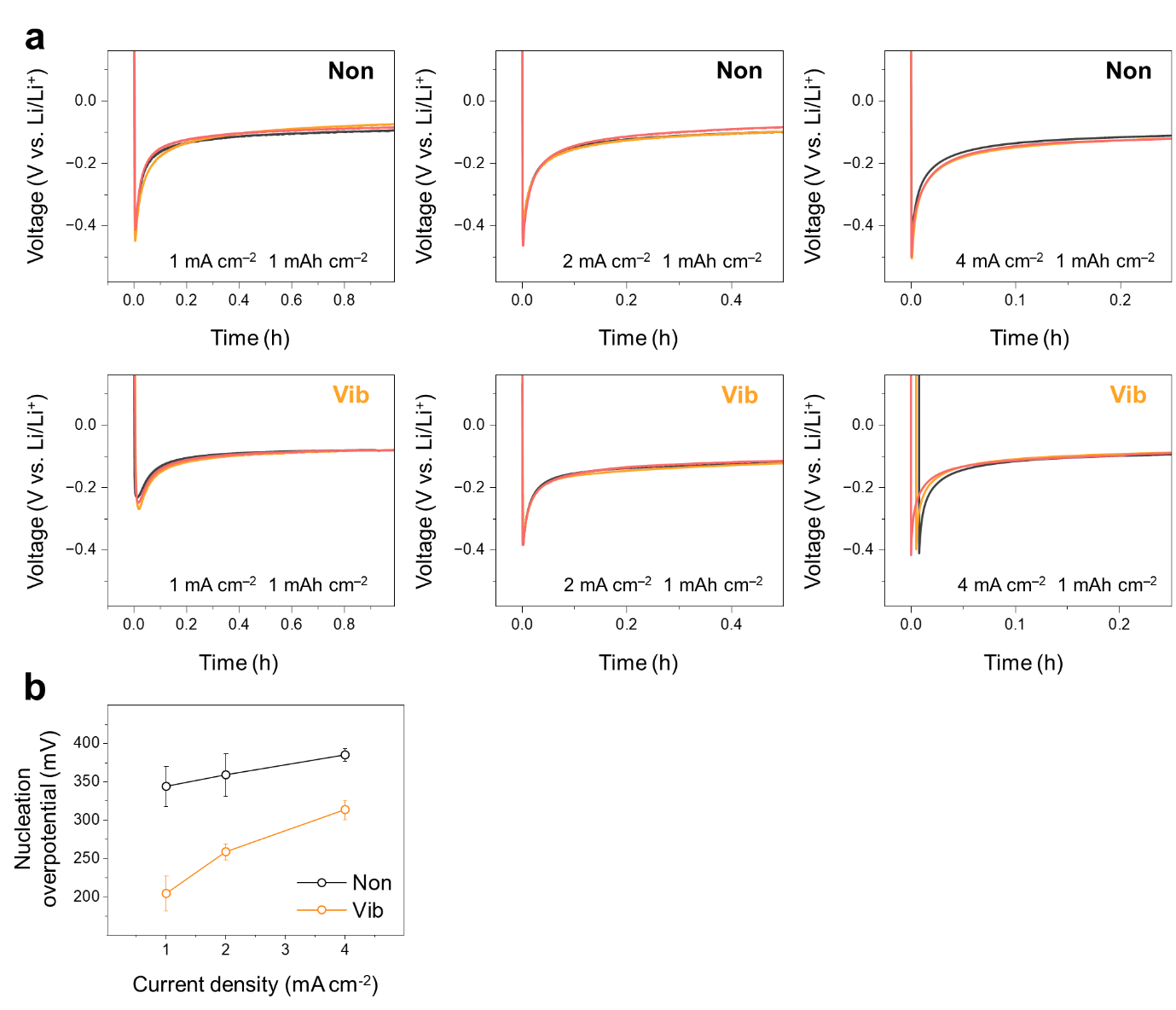


**Figure S5.** **(a)** Voltage profile of Li||Cu cells during initial Li plating on Cu electrode at various current densities, with and without vibration. (3 cells were tested for each condition to ensure reproducibility) **(b)** Corresponding nucleation overpotential extracted from the voltage profiles.

**
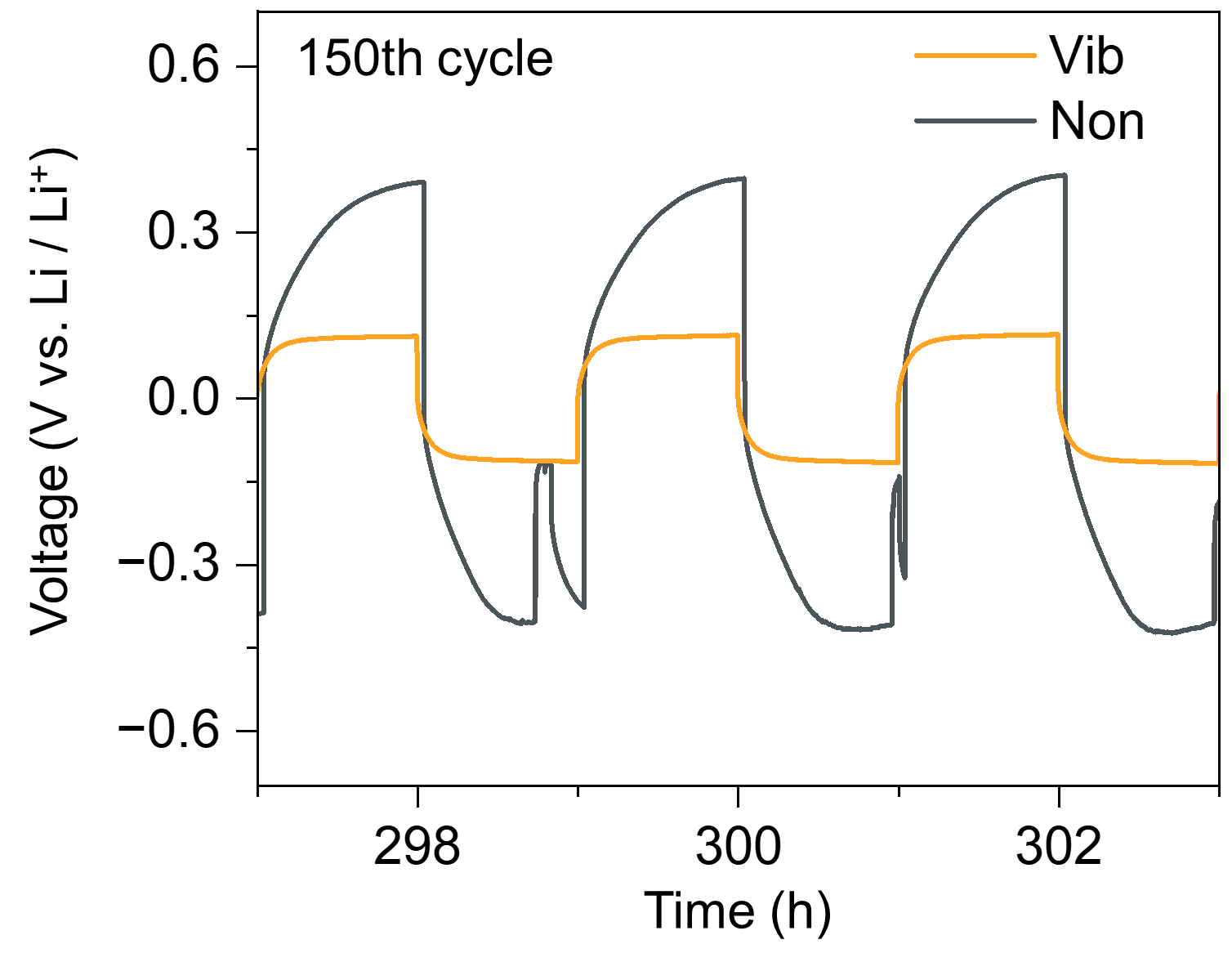
**

**Figure S6.** Voltage profiles of Li||Li symmetric cells with and without vibration at the 150th cycle.


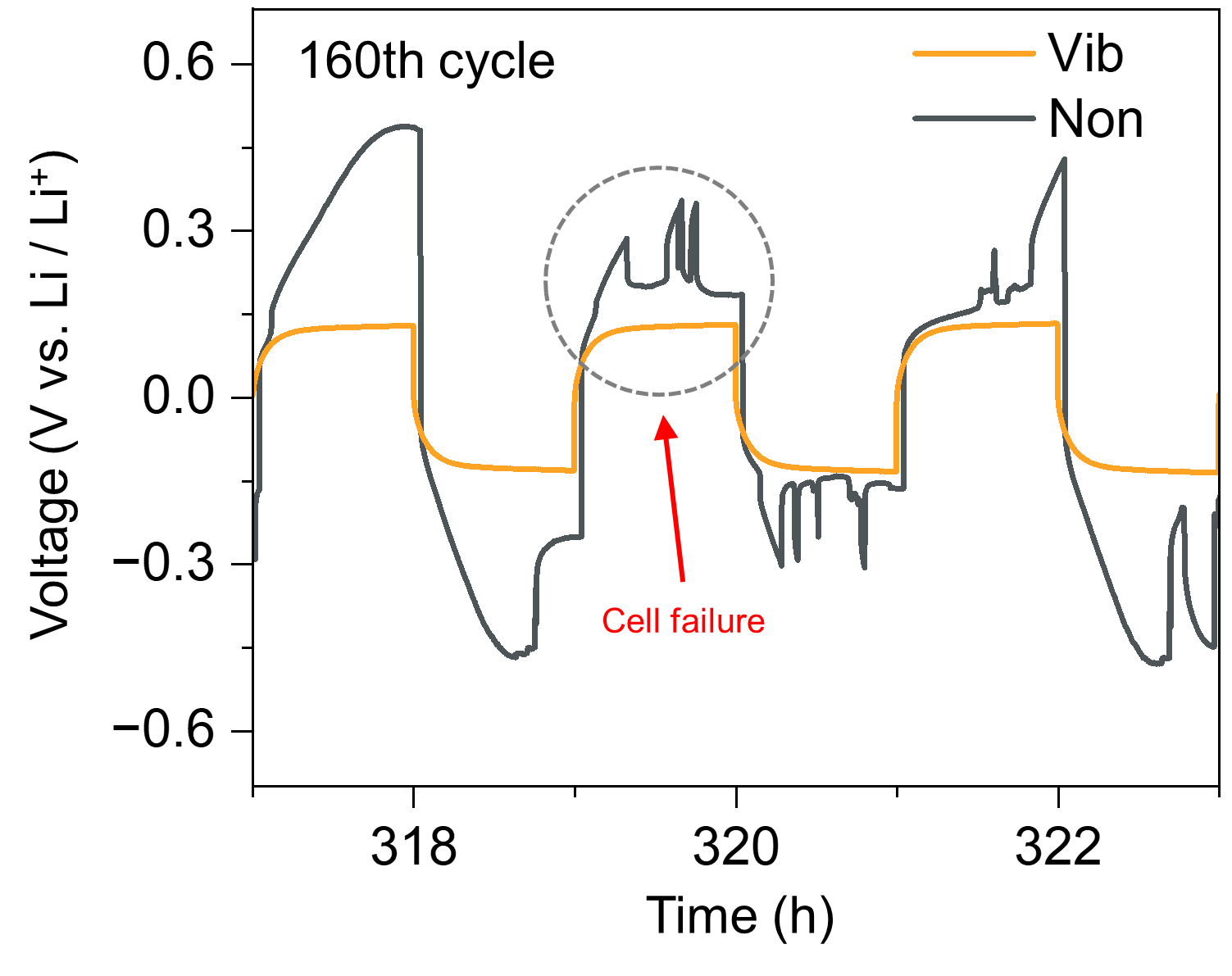


**Figure S7.** Voltage profiles of Li||Li symmetric cells with and without vibration at the 160th cycle.


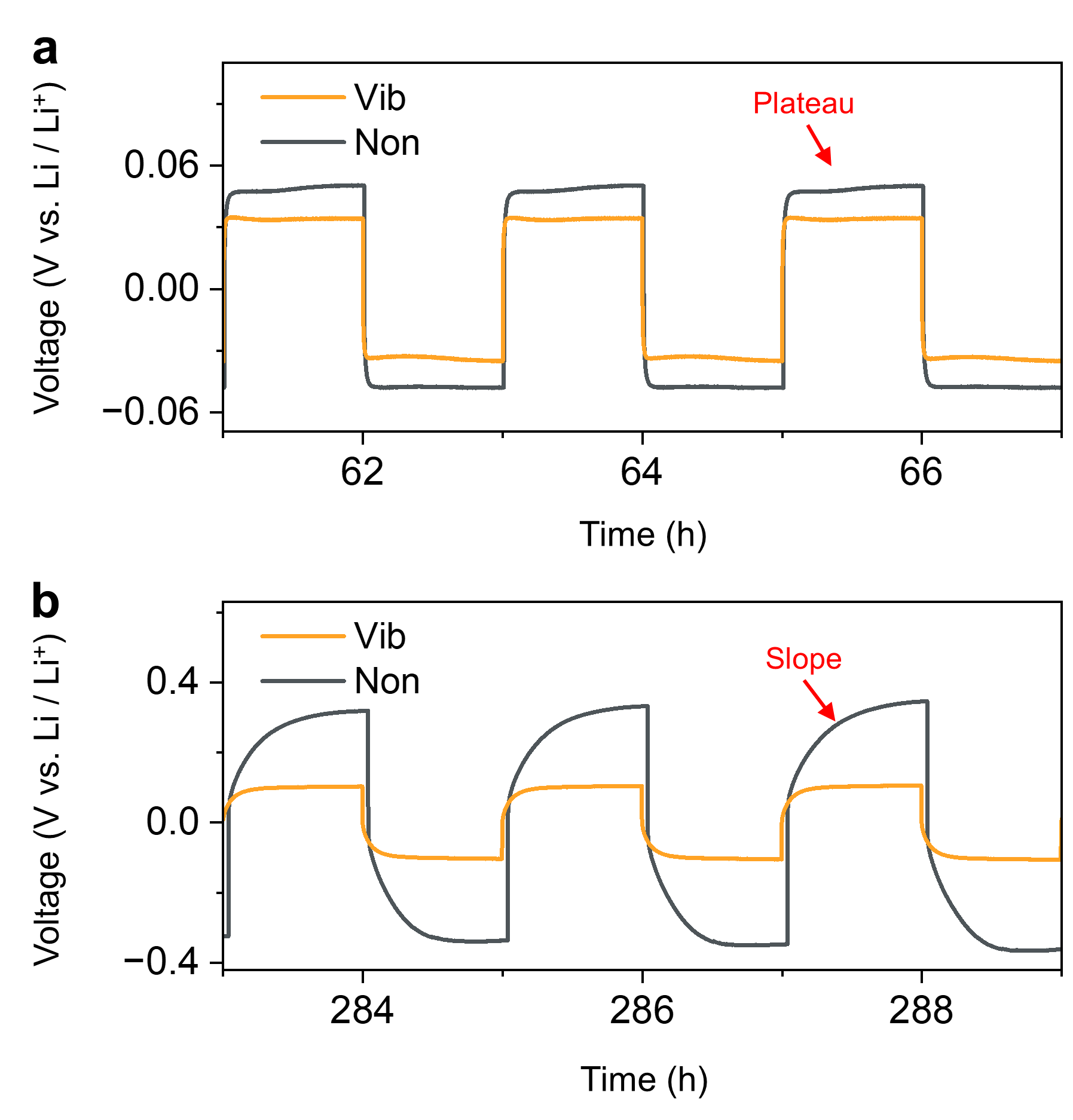


**Figure S8.** Voltage profiles of Li||Li symmetric cells with and without vibration at the **(a)** 32th and **(b)** 143th cycle.

**
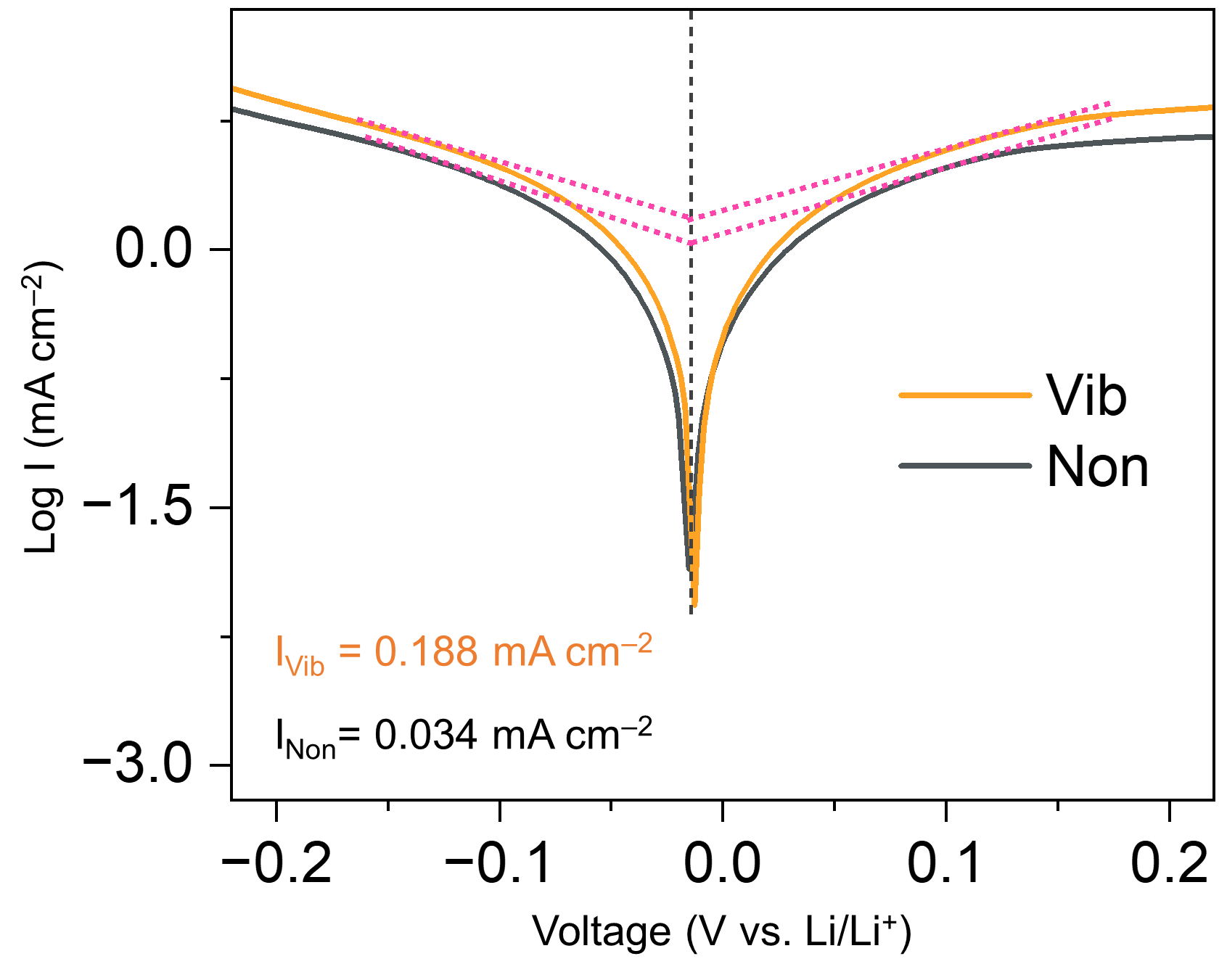
**

**Figure S9.** Tafel plots of Li symmetric cells with and without vibration after 20 cycles of cycling at a current density of 1 mA cm^–2^ with a capacity of 1 mAh cm^–2^.

**
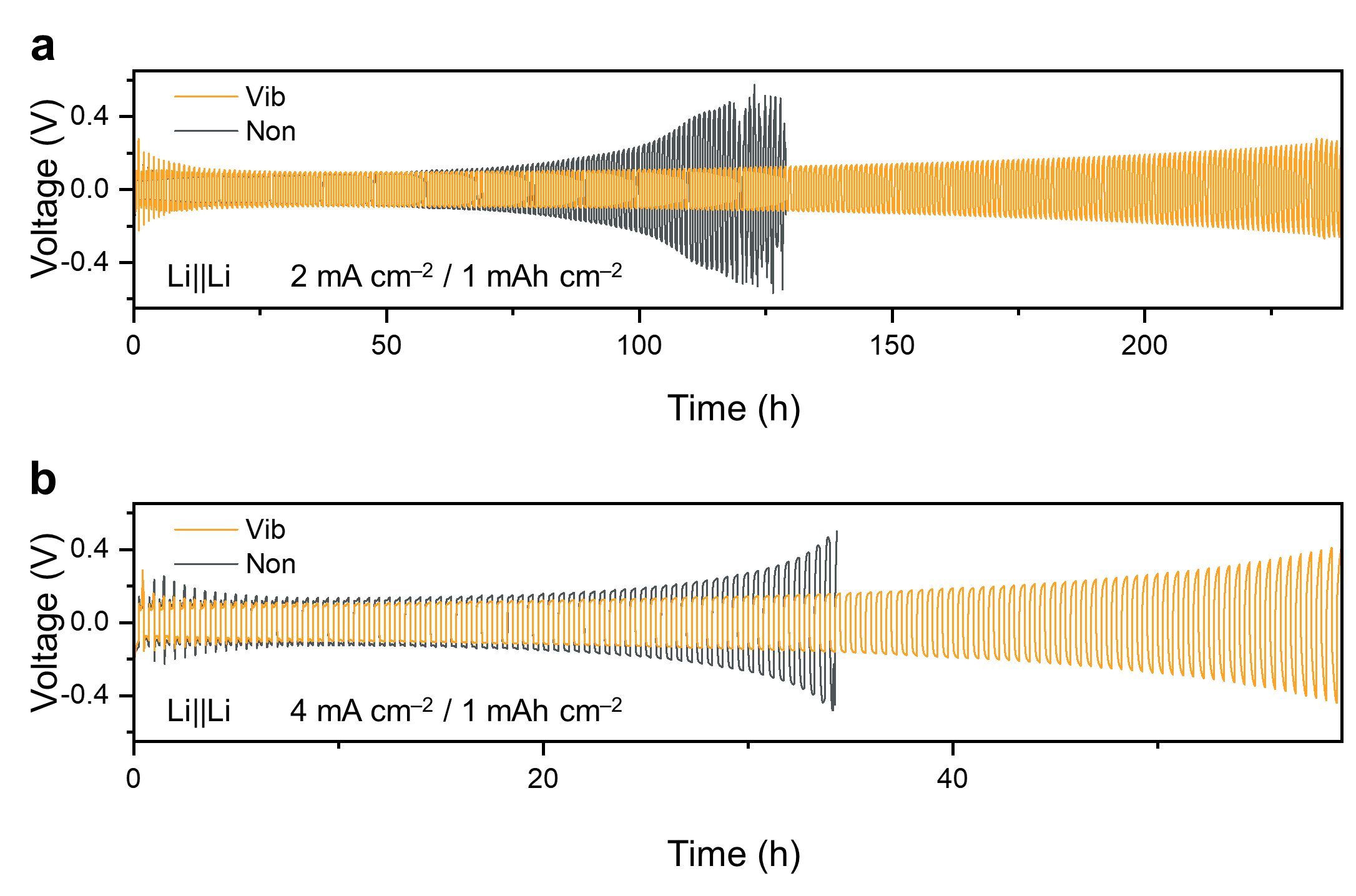
Figure S10.** The cycing performance of symmetric Li cells with and without vibration at different current densities of **(a)** 2 mA cm^–2^ and **(b)** 4 mA cm^–2^ with a capacity of 1 mAh cm^–2^.

**
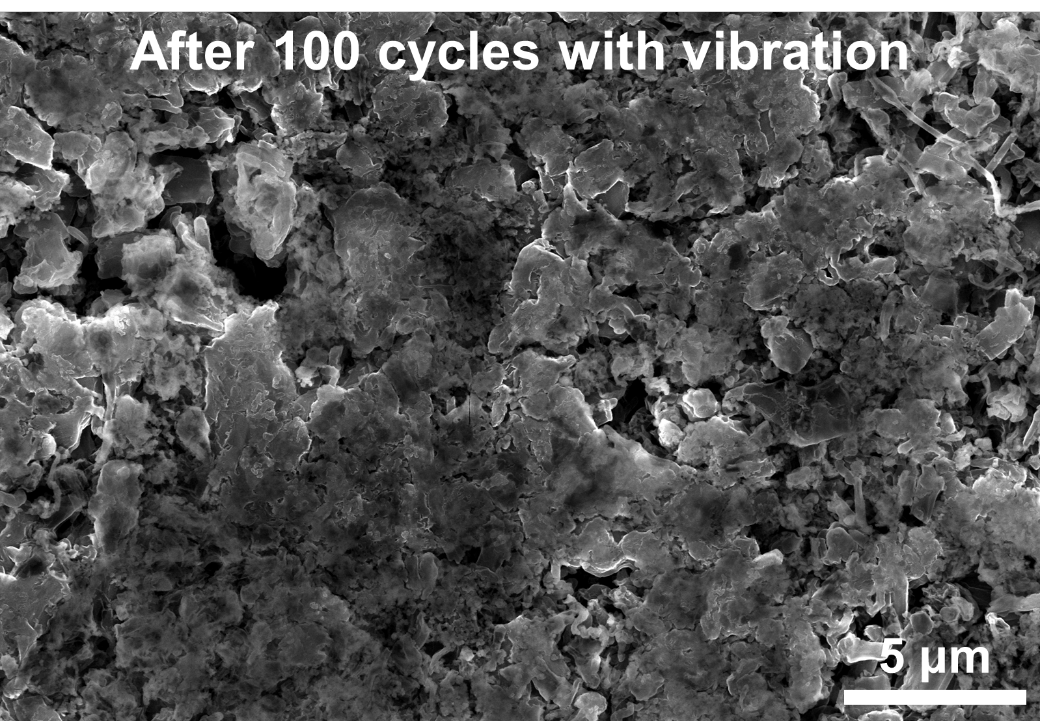
**

**Figure S11.** SEM image of Li electrode after 100 cycles at a current density of 1 mA cm^–2^ with a capacity of 1 mAh cm^–2^ with vibration.

**
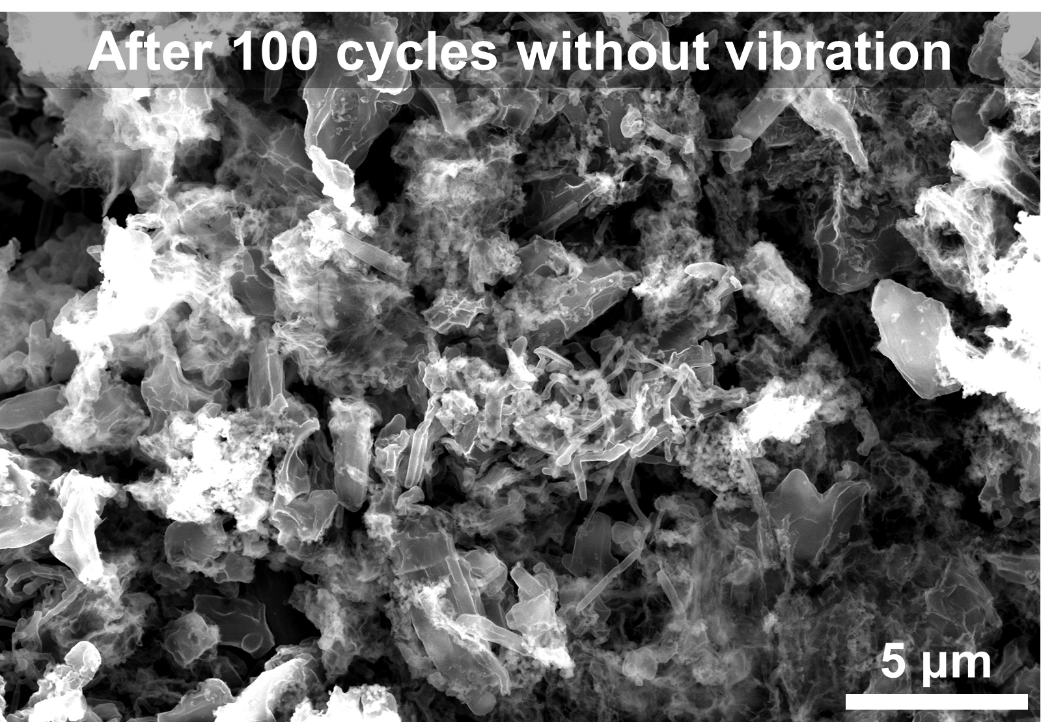
**

**Figure S12.** SEM image of Li electrode after 100 cycles at a current density of 1 mA cm^–2^ with a capacity of 1 mAh cm^–2^ without vibration.

**
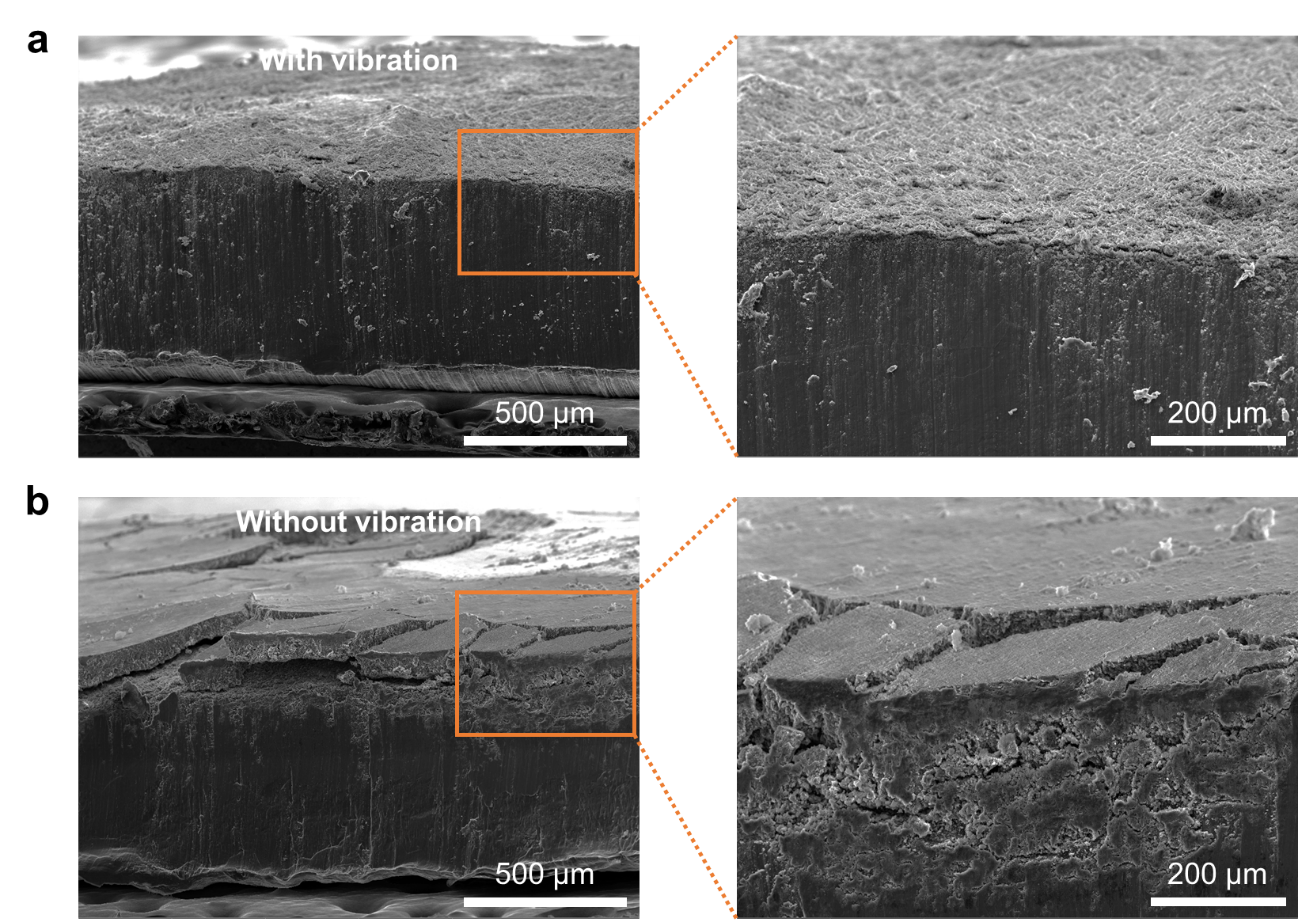
**

**Figure S13.** Cross-sectional SEM images of the Li electrode after 100 cycles **(a)** with and **(b)** without vibration at a current density of 1 mA cm^–2^ with a capacity of 1 mAh^–2^.

**
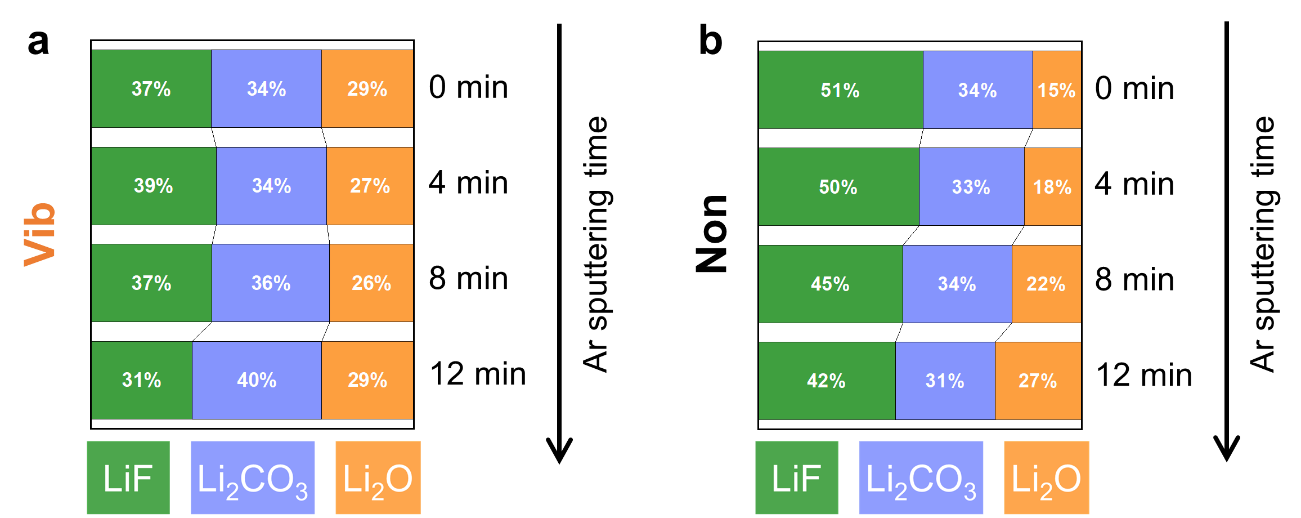
**

**Figure S14.** Relative concentration of SEI components estimated from XPS Li 1s spectra. Li cycled **(a)** with and **(b)** without vibration.

**
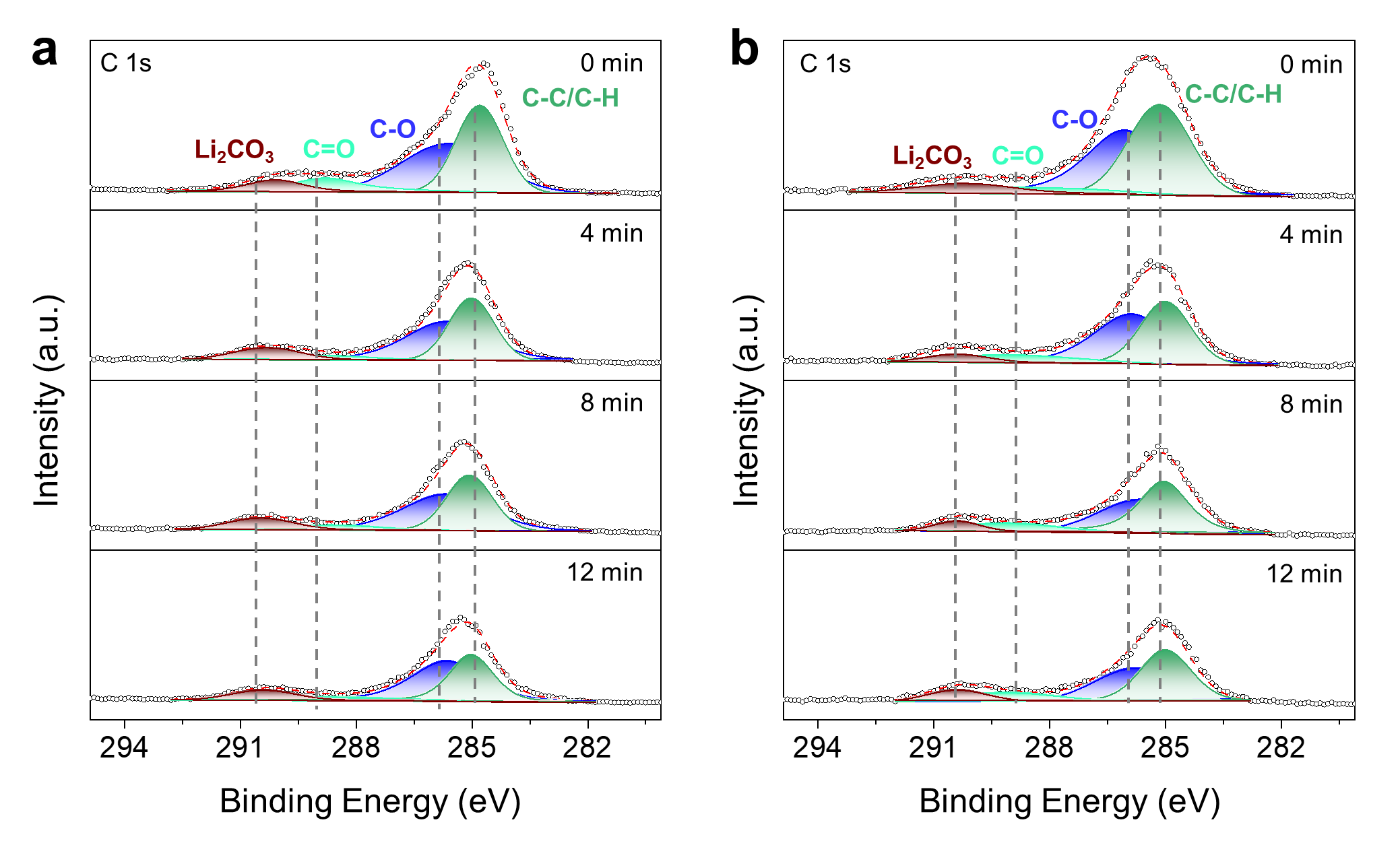
**

**Figure S15.** In-depth XPS C 1s spectra of Li electrode after 20 cycles **(a)** with and **(b)** without vibration at a current density of 1 mA cm^–2^ with a capacity of 1 mAh cm^–2^.


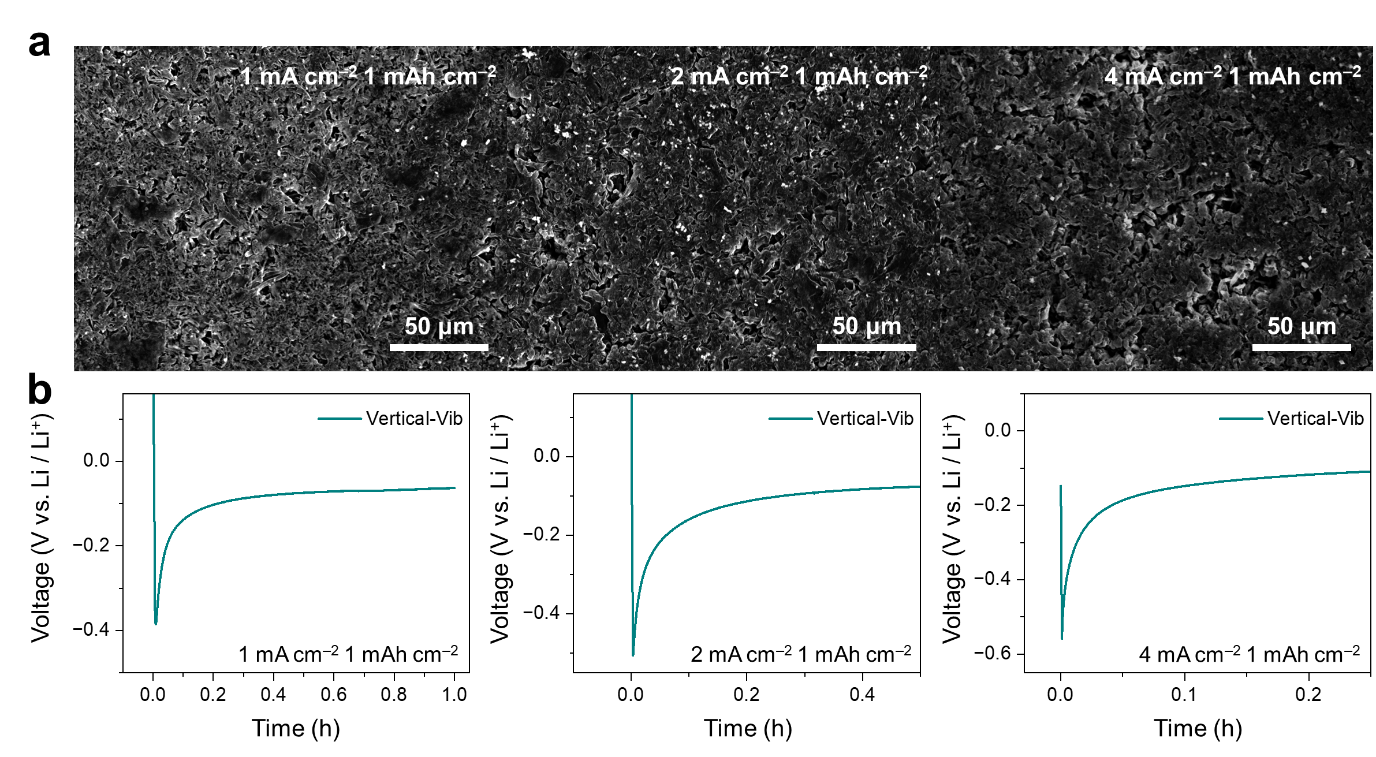
**Figure S16.** **(a)** Top-view SEM images of Li plated on Cu foil with vertical vibration at different current densities of 1 mA cm^–2^, 2 mA cm^–2^, and 4 mA cm^–2^ with an areal capacity of 1 mAh cm^–2^. **(b)** Voltage profiles during initial Li plating under vertical vibration at different current densities of 1 mA cm^–2^, 2 mA cm^–2^, and 4 mA cm^–2^ with an areal capacity of 1 mAh cm^–2^.

**
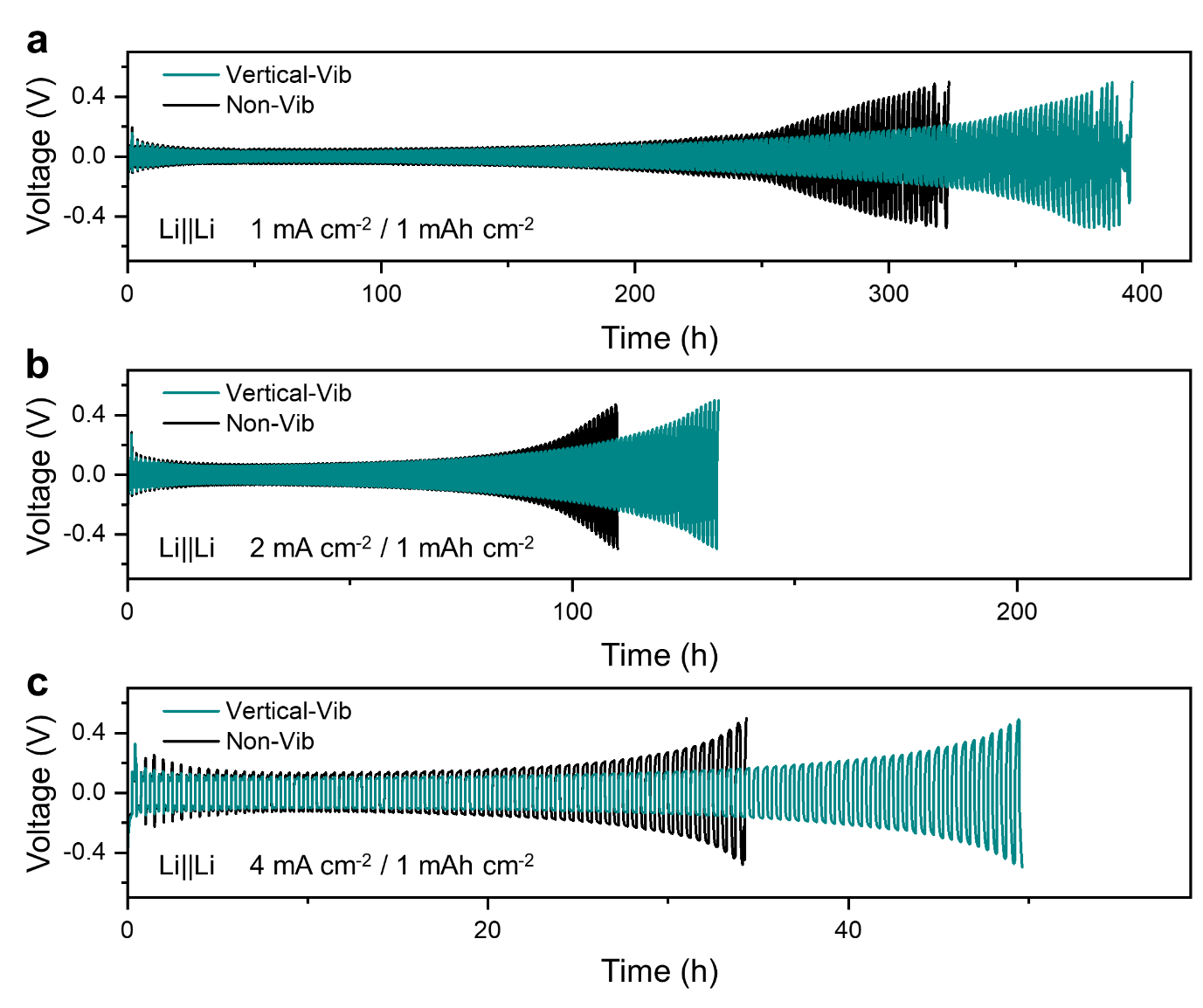
Figure S17.** The cycing performance of symmetric Li cells with vertical vibration at different current densities of **(a)** 1 mA cm^–2^, **(b)** 2 mA cm^–2^, and **(c)** 4 mA cm^–2^ with a capacity of 1 mAh cm^–2^.

**
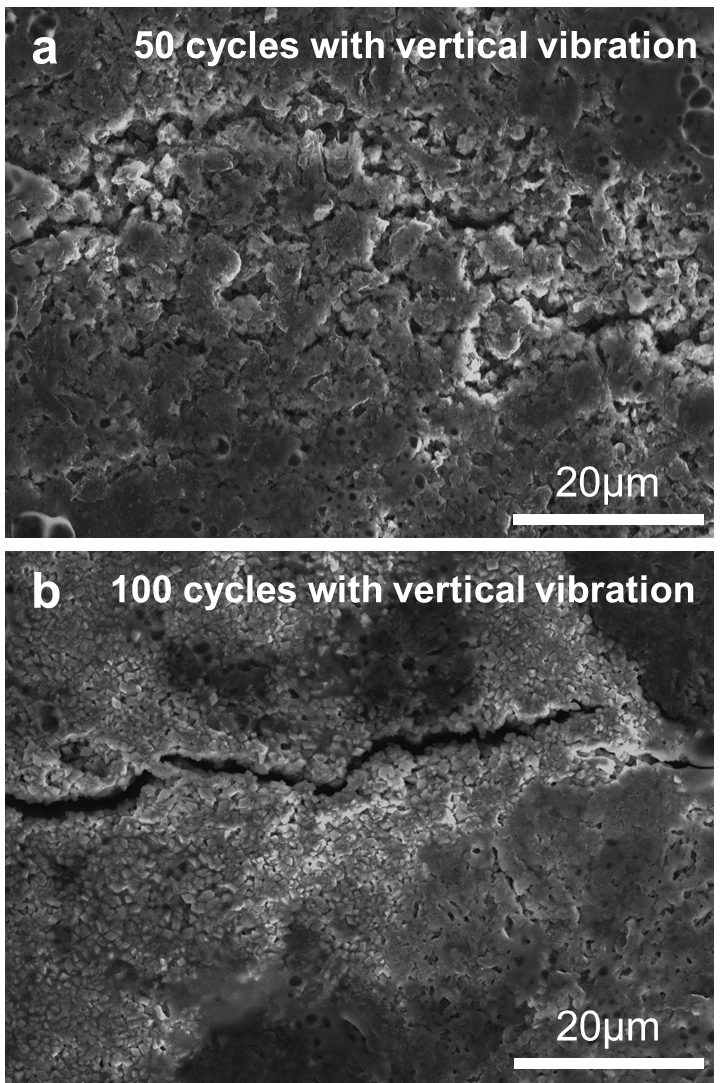
**

**Figure S18.** Top-view SEM images of Li electrode after **(a)** 50, and **(b)** 100 cycles with vertical vibration at a current density of 1 mA cm^–2^ with a capacity of 1 mAh cm^–2^.


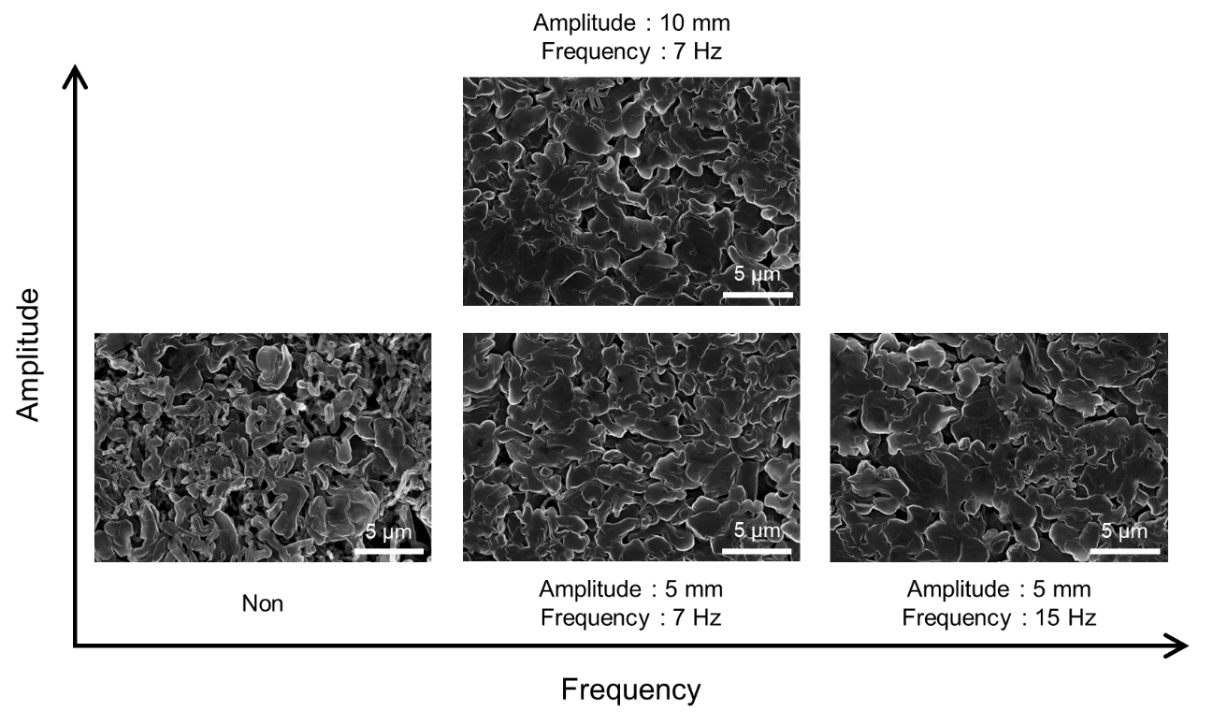


**Figure S19.** SEM images of plated Li after deposited on Cu at a current density of 1 mA cm^-2^ with a deposition capacity of 1mAh cm^-2^ under different vibration conditions.


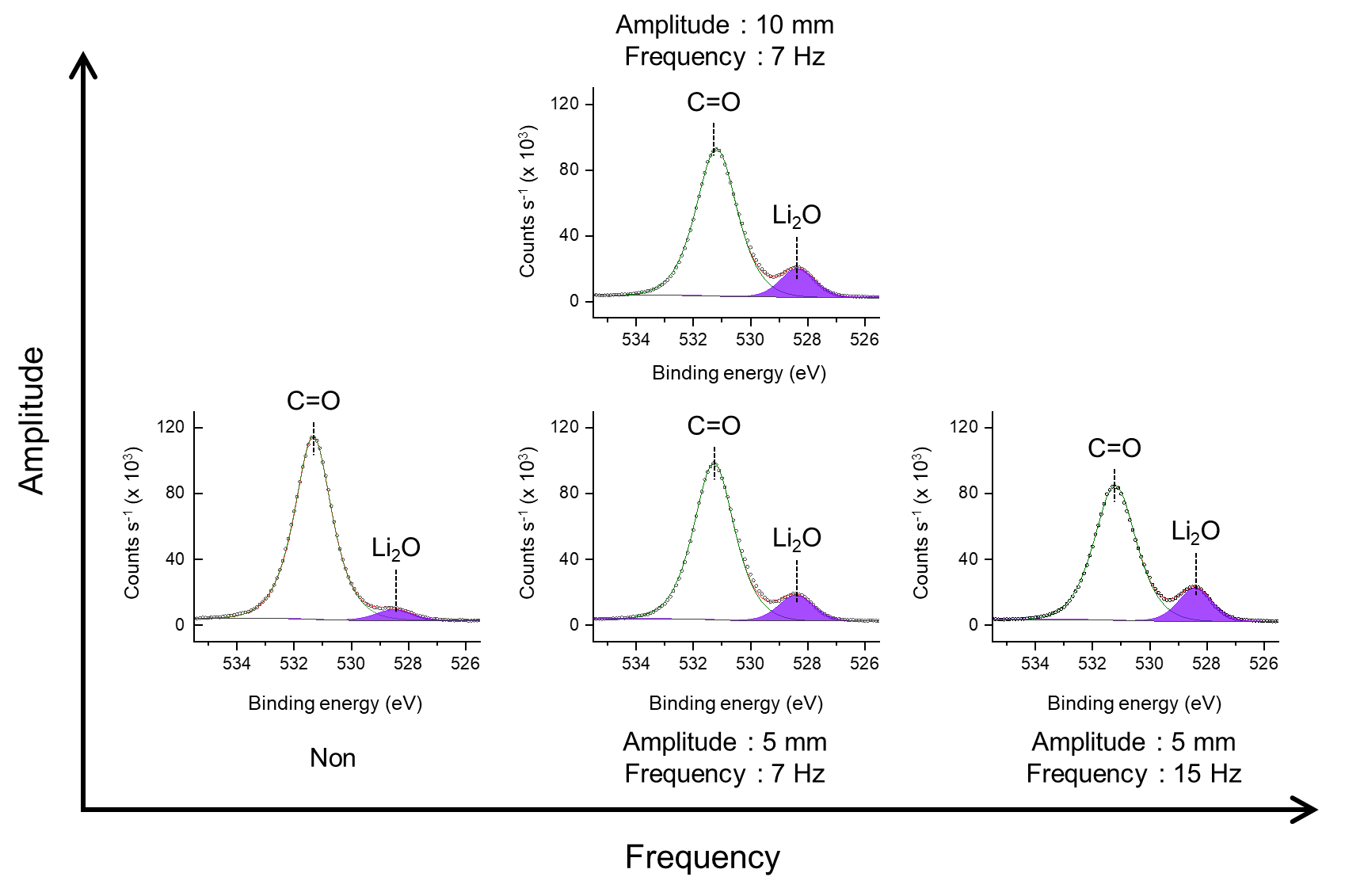


**Figure S20.** XPS O1s spectra of Li after deposited on Cu at a current density of 1 mA cm^-2^ with a deposition capacity of 1mAh cm^-2^ under different vibration conditions.

**
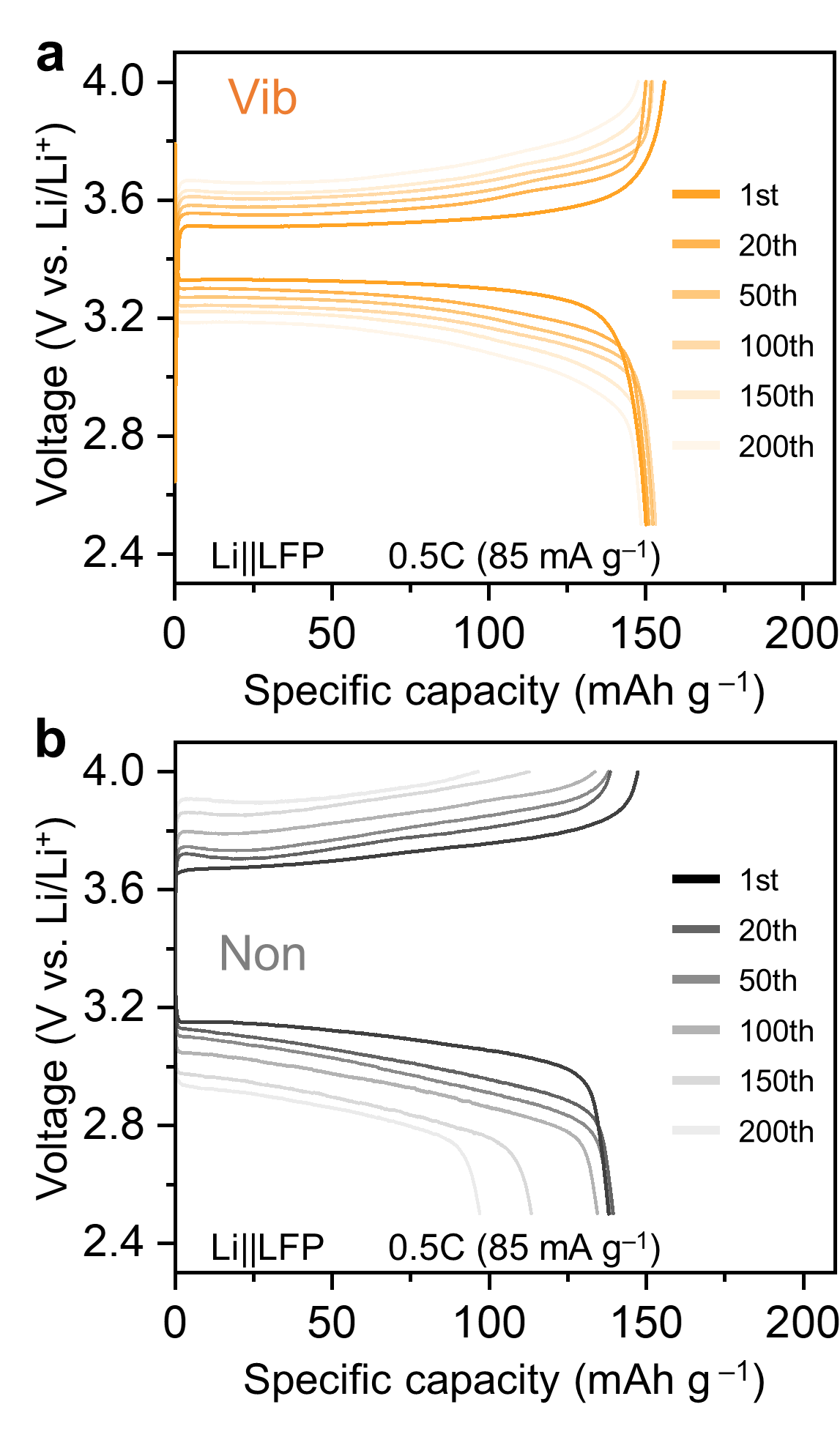
**

**Figure S21.** Voltage profiles of Li||LFP cells cycled at 0.5 C **(a)** with and **(b)** without vibration during the 1st, 20th, 50th, 100th, 150th, and 200th cycles.

**
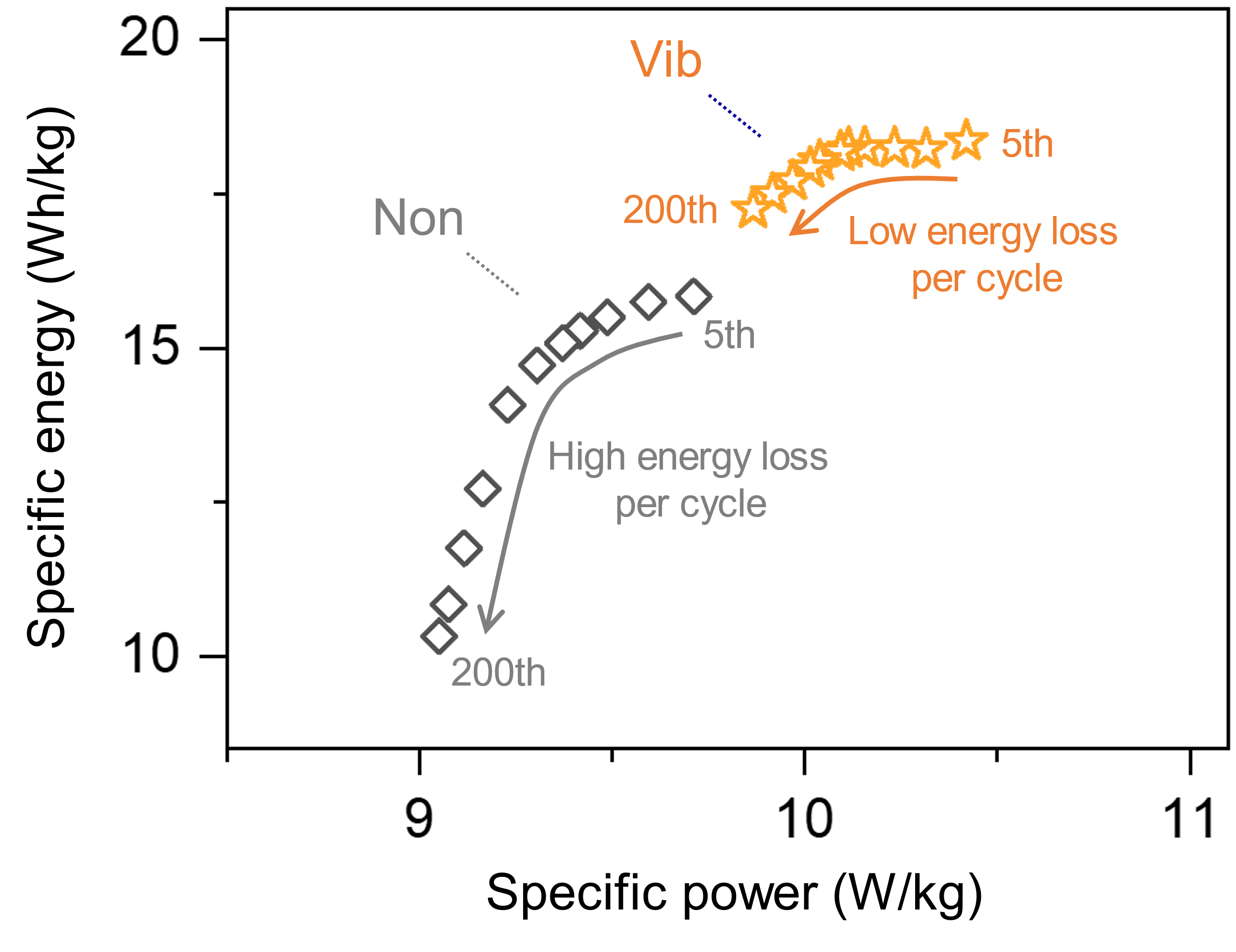
**

**Figure S22.** Ragone plot comparing Li||LFP cells cycled at 0.5 C (85 mA g^–1^) with and without vibration, based on selected cycles (5th, 20th, 40th, 60th, 80th, 100th, 120th, 140th, 160th, 180th, and 200th cycles). Specific energy and power of the cells were calculated based on the total weight of the anode, cathode, electrolyte, and separator.

**
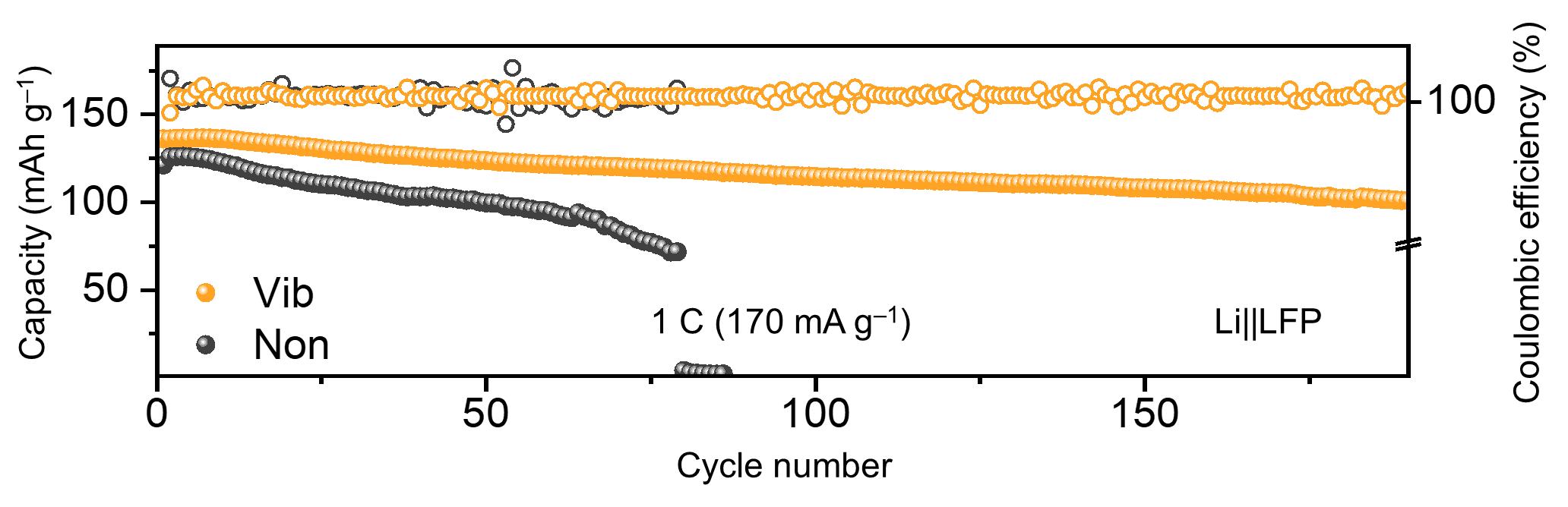
**

**Figure S23.** Cycling performance of the Li||LFP cell as a function of cycle number in the voltage range of 2.5–4.0 V at 1 C (170 mA g^–1^).


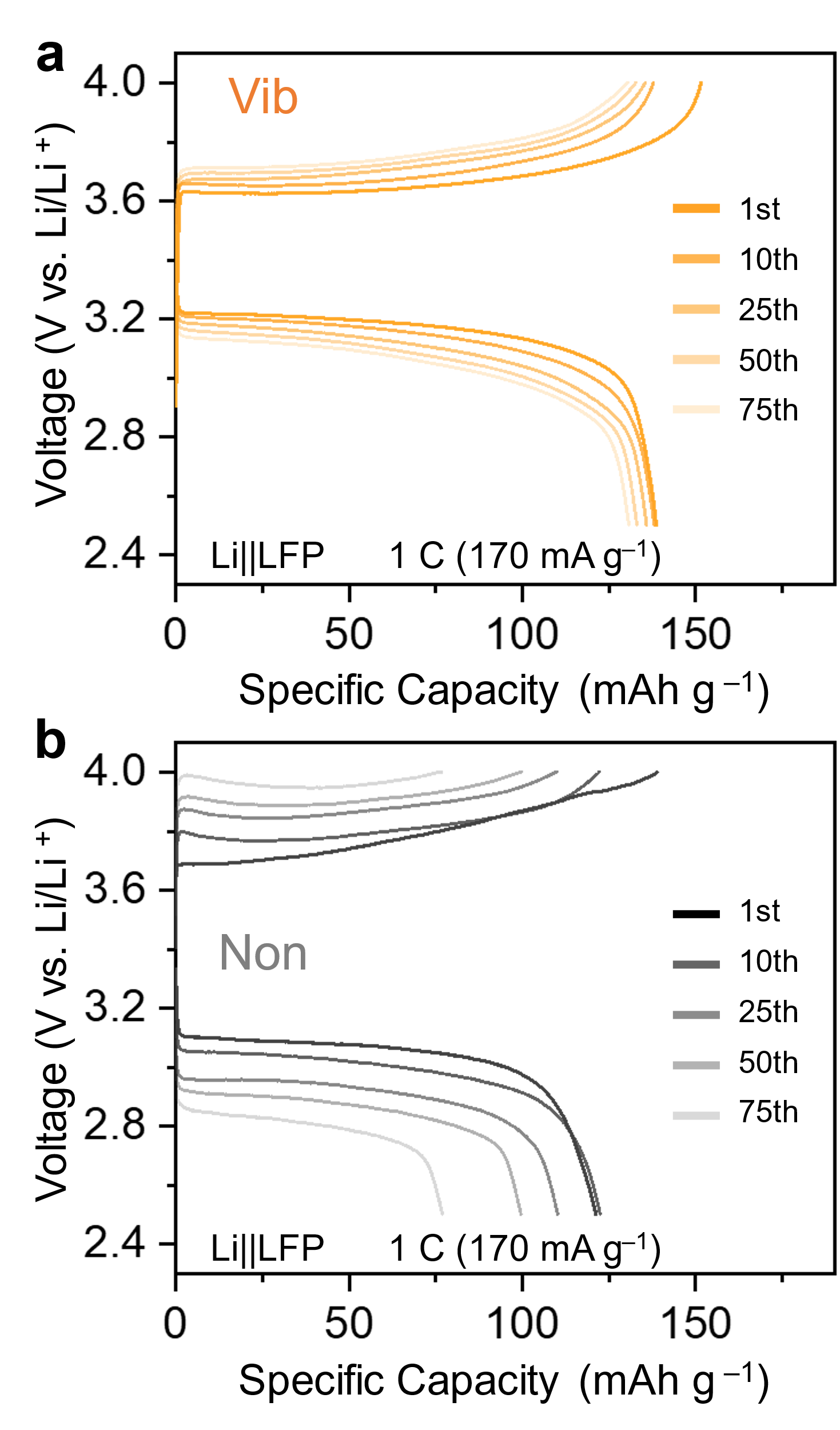


**Figure S24.** Voltage profiles of Li||LFP cells cycled at 1 C **(a)** with and **(b)** without vibration during the 1st, 10th, 25th, 50th, and 75th cycles.


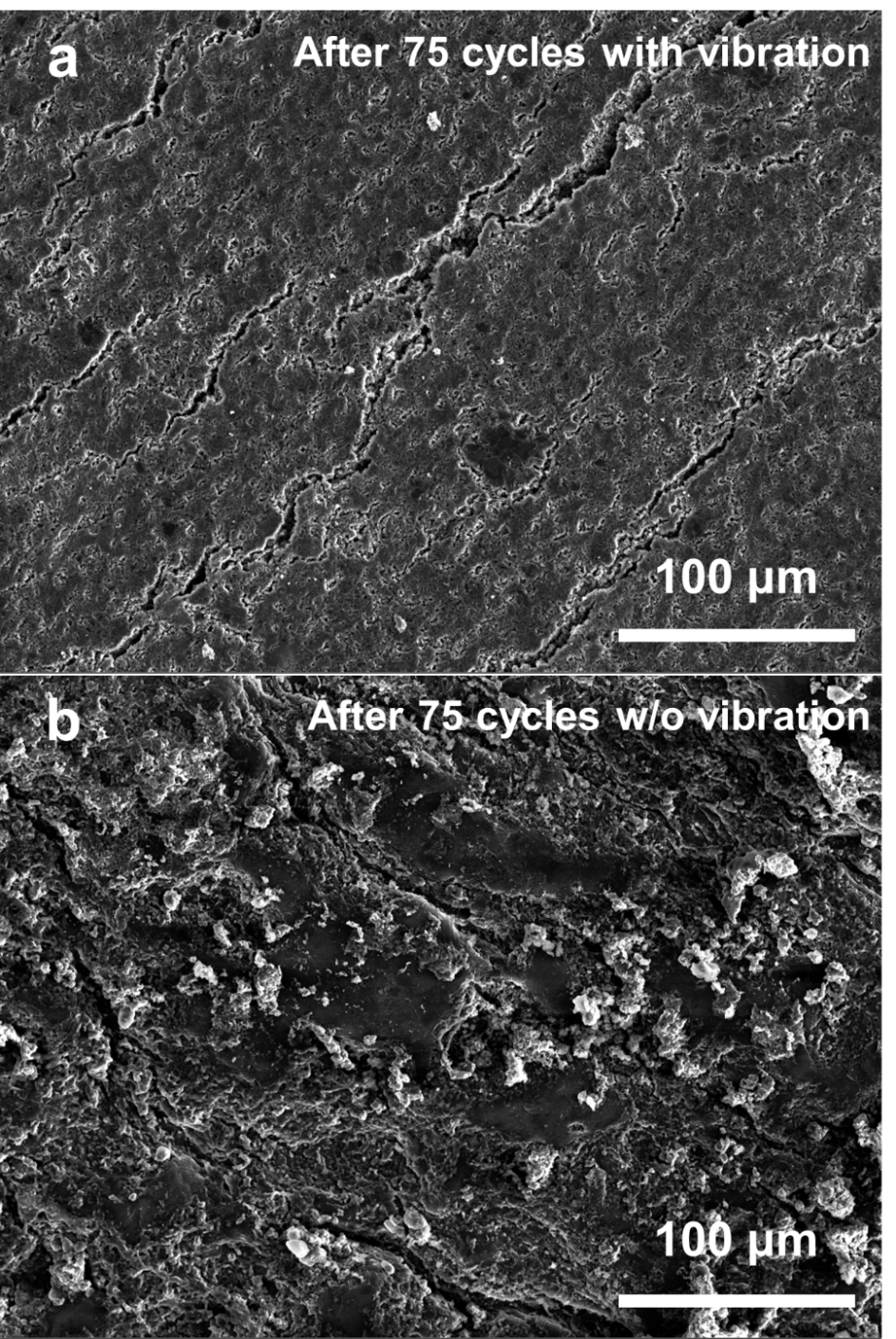


**Figure S25.** High-magnification SEM images of Li electrodes after 75 cycles at 1C **(a)** with and **(b)** without vibration.

**
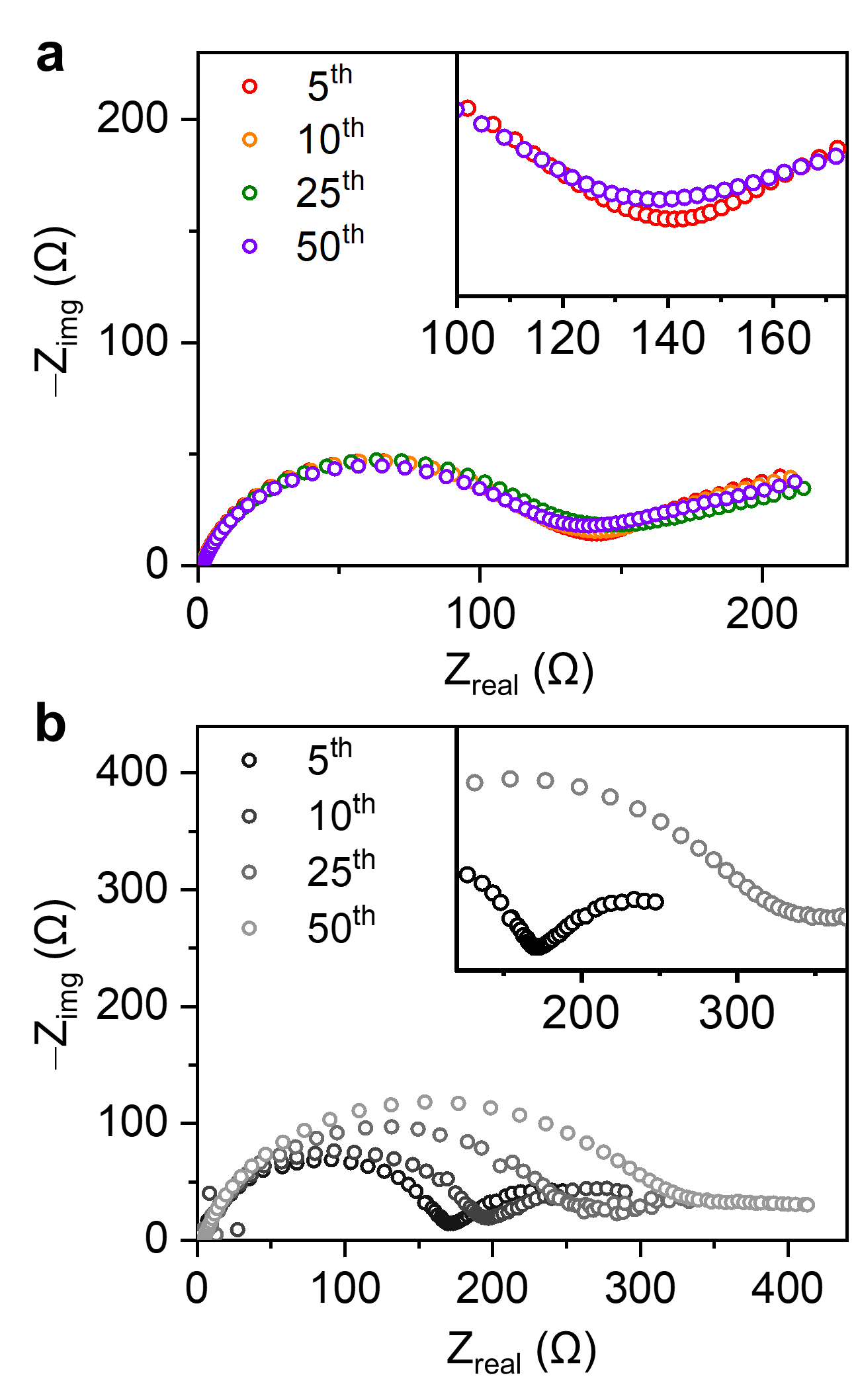
**

**Figure S26.** Nyquist plots of Li||LFP cells cycled **(a)** with and **(b)** without vibration at selected cycles (5th, 10th, 25th, and 50th cycles).

**
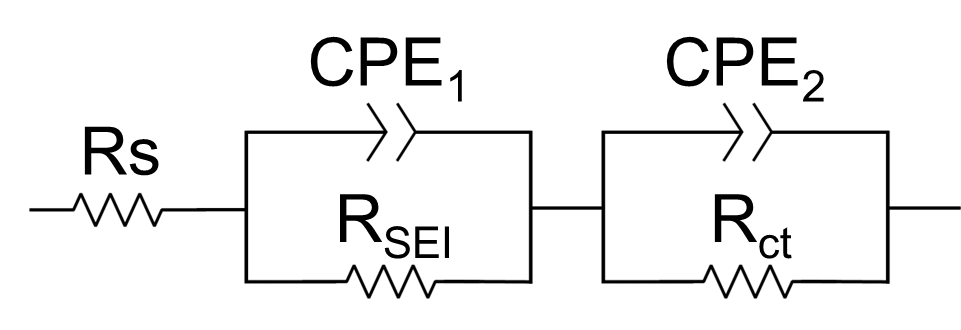
**

**Figure S27.** Equivalent circuit for modelling Nyquist plots of Li||LFP cells.

**Table S1.** Fitted results of Li||LFP cells with and without vibration after the 5^th^, 10^th^,

25^th^, and 50^th^ cycle.

| Sample | R_s_ (Ω) | R_Interface_ (Ω) |
| --- | --- | --- |
| Horizontal_5^th^ | 1.37 | 141.93 |
| Non-Vib_5^th^ | 2.21 | 205.39 |
| Horizontal_10^th^ | 1.52 | 145.76 |
| Non-Vib_10^th^ | 3.05 | 235.7 |
| Horizontal_25^th^ | 1.71 | 153.63 |
| Non-Vib_25^th^ | 4.09 | 232.05 |
| Horizontal_50^th^ | 1.83 | 149.66 |
| Non-Vib_50^th^ | 4.43 | 356.02 |

**
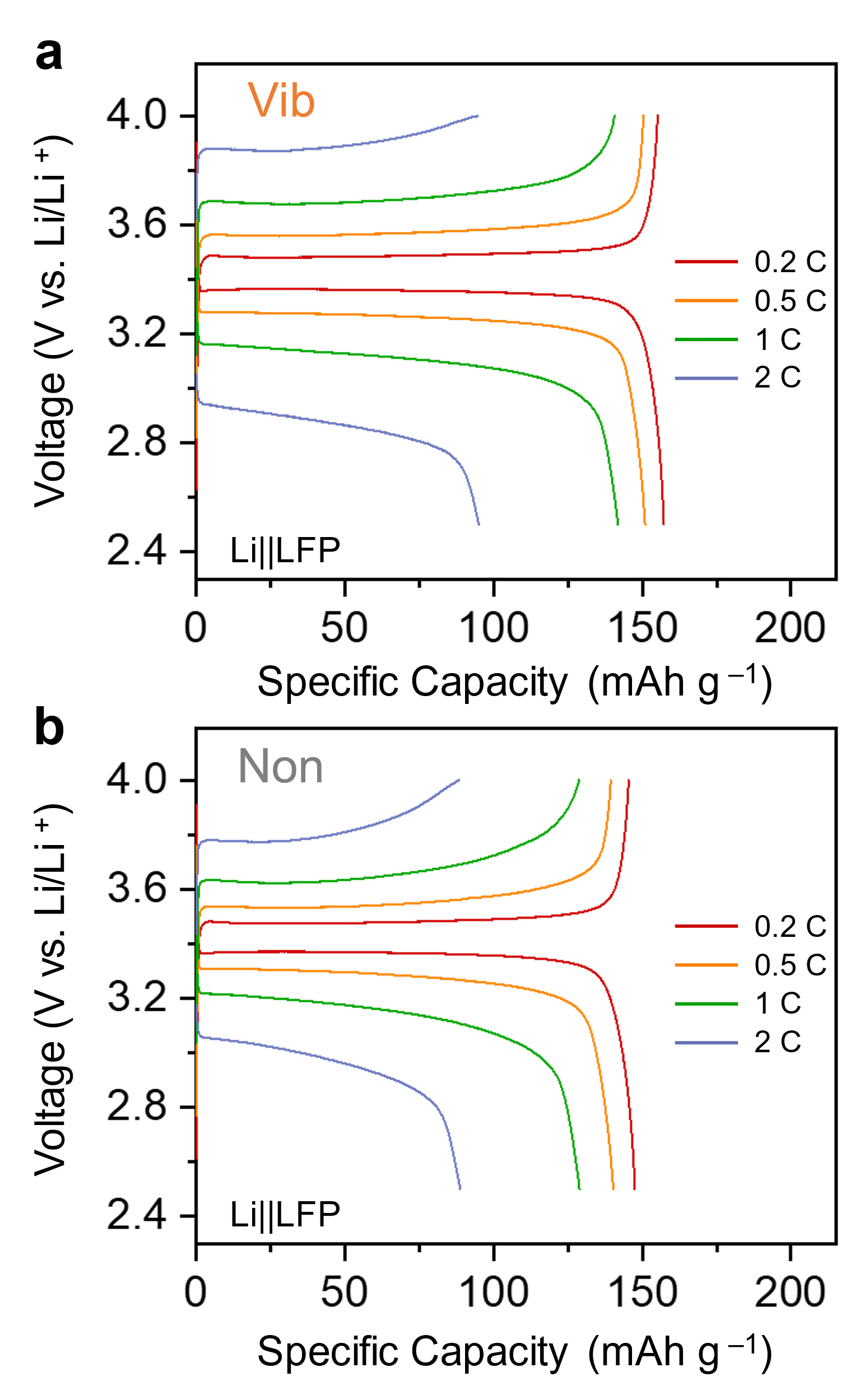
**

**Figure S28.** Rate capability of Li||LFP cells **(a)** with and **(b)** without vibration at current densities of 0.2 C, 0.5 C, 1 C, and 2 C. The C-rate is based on the capacity of the LFP cathode (1C = 170 mAh g^–1^).

**
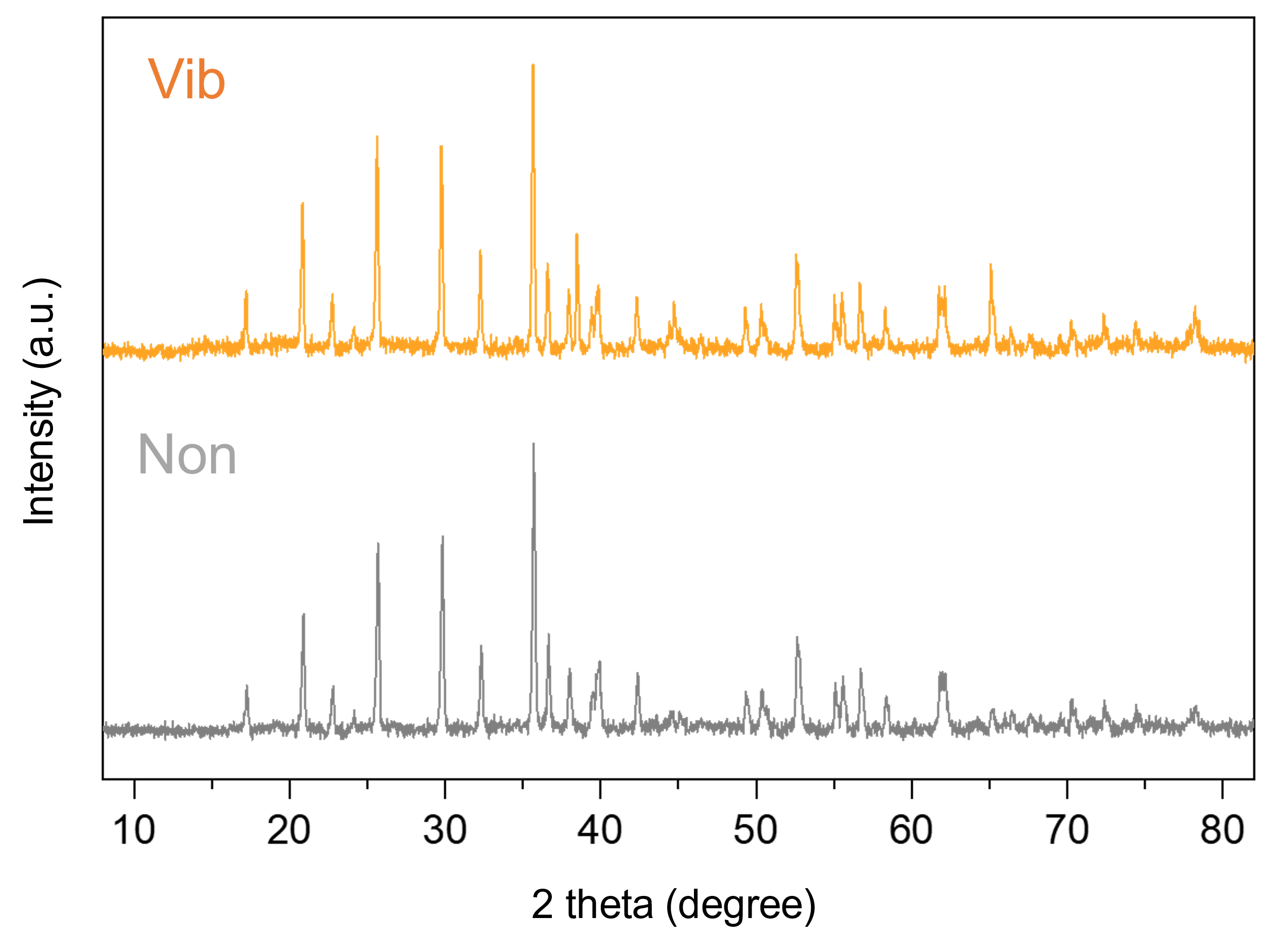
**

**Figure S29.** X-ray diffraction (XRD) comparing LFP cathodes retrieved after 75 cycles with and without vibration.

**
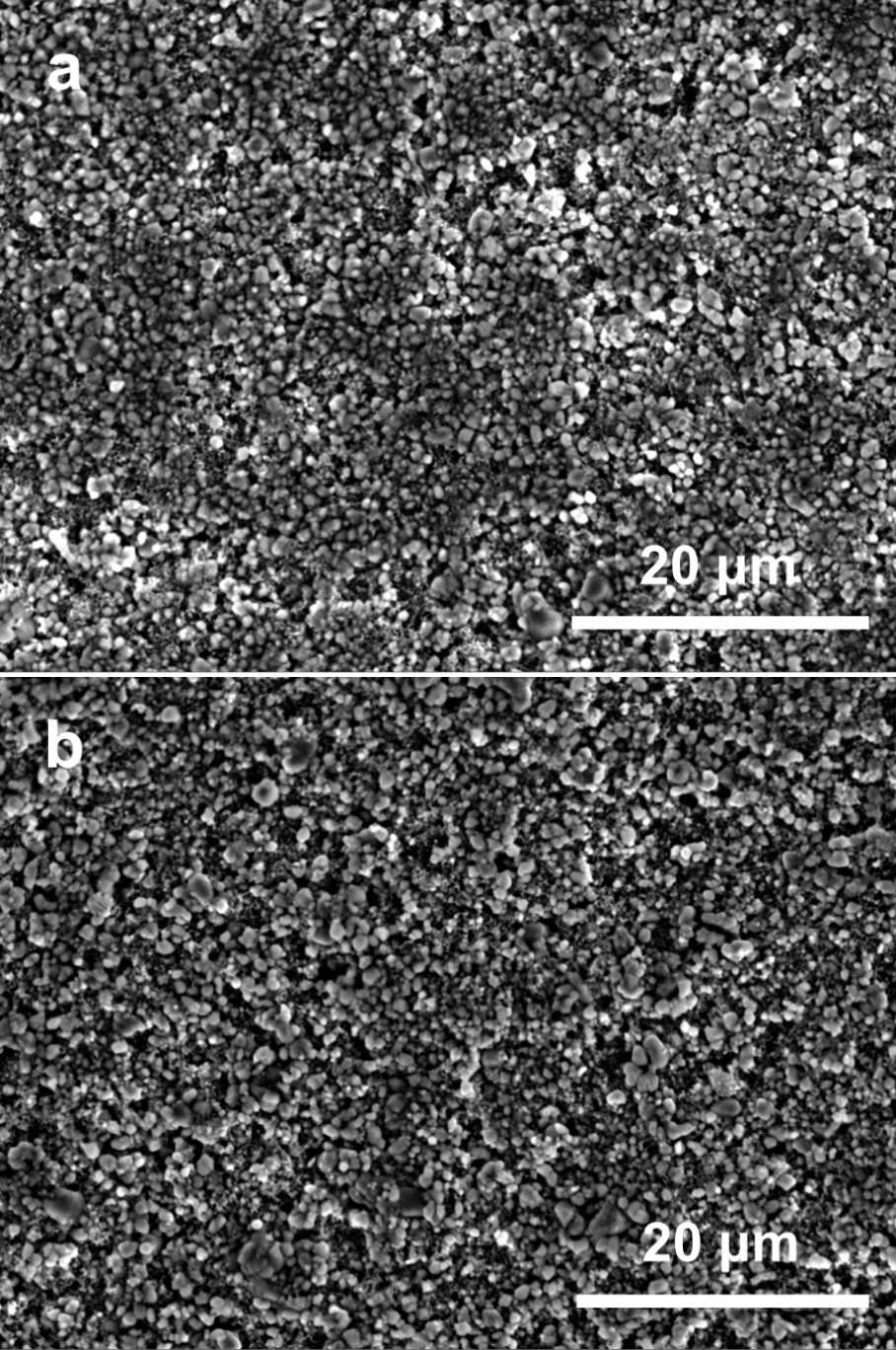
**

**Figure S30.** Top-view SEM images of LFP electrode obtained from cycled Li||LFP cells after 100 cycles **(a)** with and **(b)** without vibration at a current density of 0.5 C (170 mAh g^–1^).

**
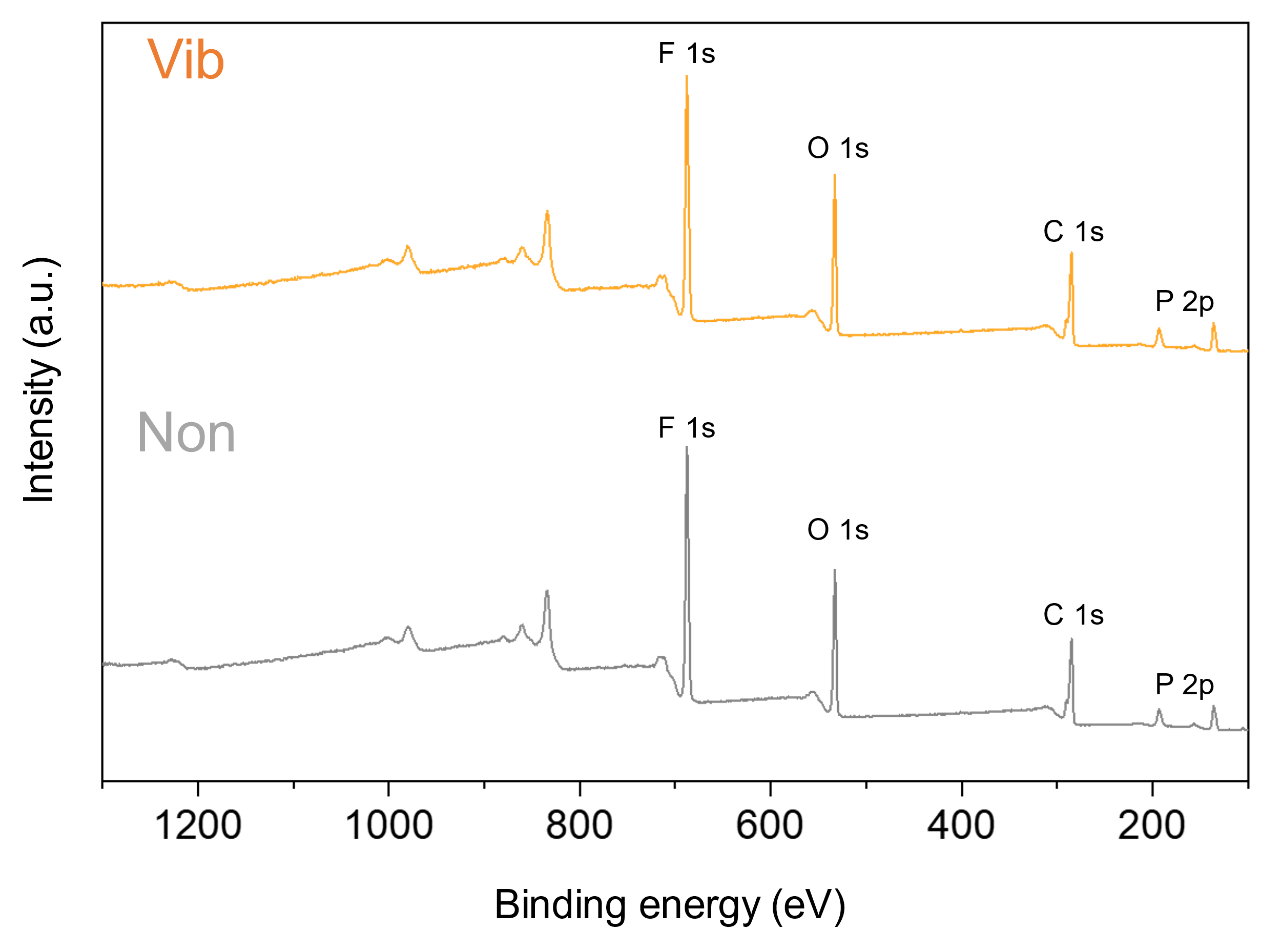
**

**Figure S31.** XPS spectra comparing LFP cathodes retrieved after 75 cycles with and without vibration.

**
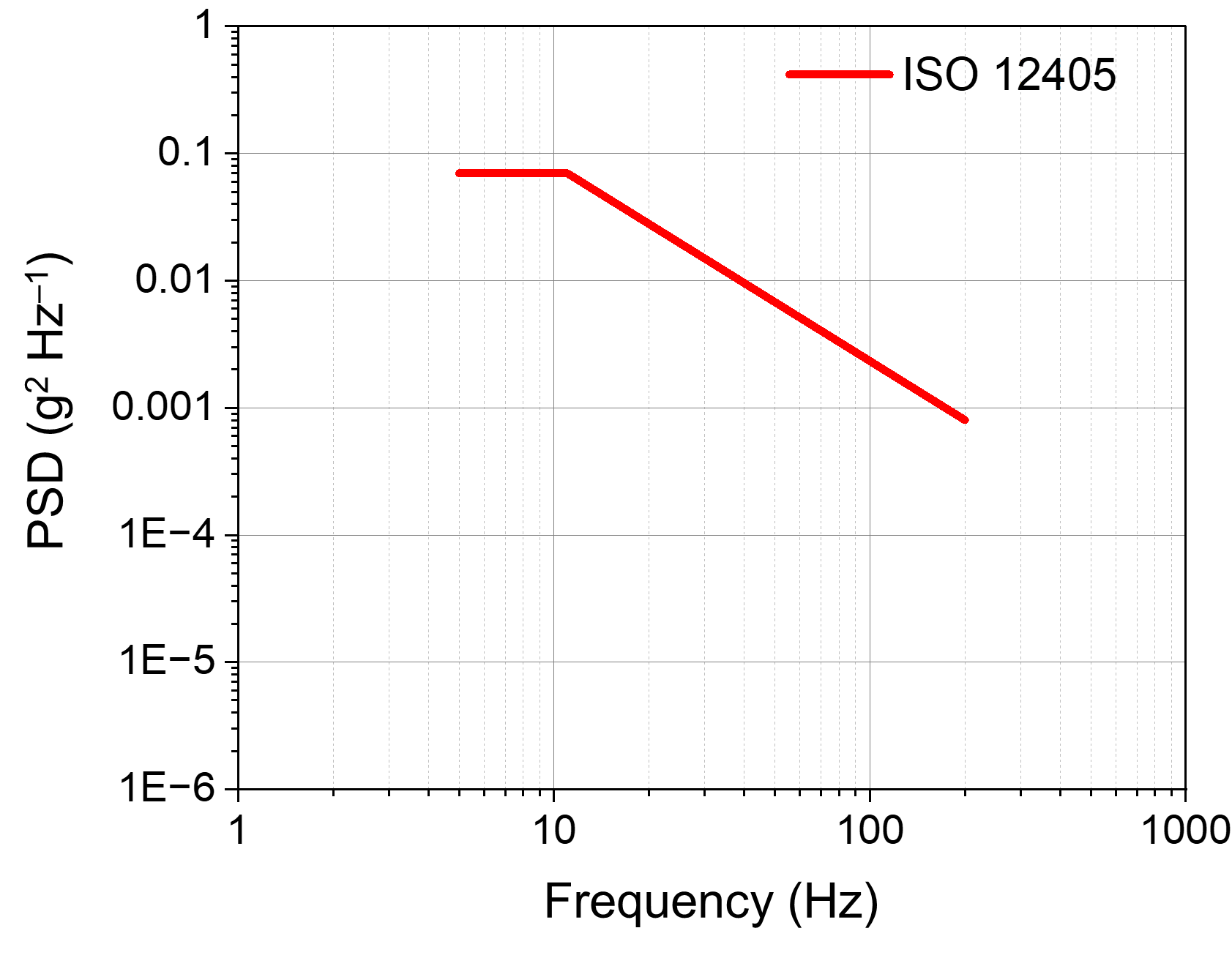
**

**Figure S32.** The vibration test profile for battery in EVs (ISO 12405).

**
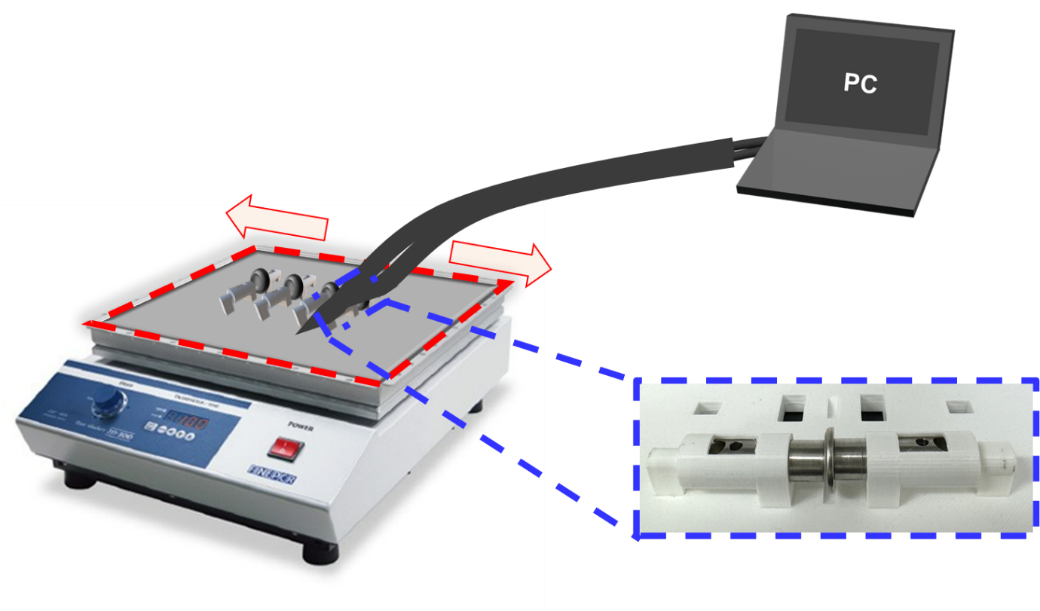
**

**Figure S33.** A schematic illustrating the imposition of vibration on the cells.
